# Noncovalent Interactions in Density Functional Theory: All the Charge Density We Do Not See

**DOI:** 10.1021/jacs.5c13706

**Published:** 2025-10-23

**Authors:** Almaz Khabibrakhmanov, Matteo Gori, Carolin Müller, Alexandre Tkatchenko

**Affiliations:** Department of Physics and Materials Science, 112680 University of Luxembourg, L-1511 Luxembourg City, Luxembourg

## Abstract

Exact determination of the electronic density of molecules and materials would provide direct access to accurate bonded and nonbonded interatomic interactions via the Hellman–Feynman theorem. However, density-functional approximations (DFAs)—the workhorse methods for the electronic structure of atomistic systems—only provide approximate and sometimes unreliable electron densities. In this work, we demonstrate that long-range van der Waals (vdW) dispersion interactions can induce significant polarization in the electron density, with the magnitude of effect growing with system size. We evaluate vdW-induced density shifts using newly developed fully coupled and optimally tuned variant of many-body dispersion model (MBD@FCO), benchmarked against accurate coupled-cluster densities. Applied to supramolecular data sets (S12L and L7) and a prototype protein (Fip35-WW), our approach reveals that dispersion-driven polarization alters long-range electrostatic potentials by up to 4 kcal/mol and reshapes noncovalent interaction (NCI) isosurfaces, producing smooth and chemically interpretable interaction regions. These findings demonstrate that dispersion interactions leave a measurable imprint on the electron density, with implications for electrostatics, biomolecular modeling, and density-based chemical analysis. Our results bridge energy-based dispersion models and density-functional theory, paving the way toward dispersion-consistent DFAs and improved machine-learned models based on electron densities.

## Graphical abstract

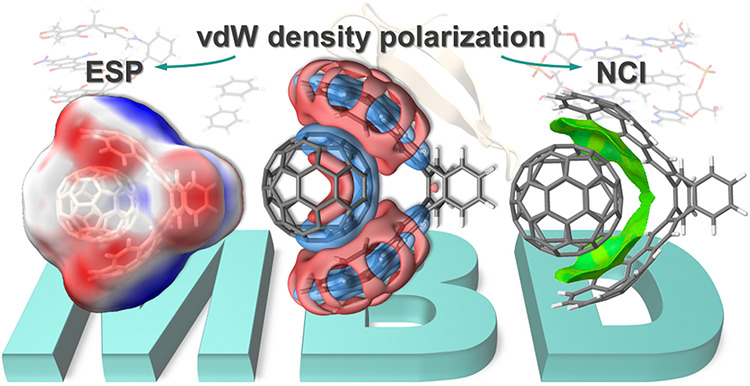

## Introduction

The electronic charge density ρ­(**r**) is a fundamental observable that uniquely defines molecules and materials with nondegenerate electronic ground states.^1,2^ It plays a central role in density-functional approximations (DFAs), which are widely used in computational chemistry and materials science. Existing DFAs often produce reasonable approximations to the ground state energy, which makes them so useful in practice. However, the accuracy of DFAs in predicting energies has not necessarily translated into better predictions of ρ­(**r**). This trade-off was highlighted in the seminal work by Medvedev et al.,^3^ revealing discrepancies between DFA-generated densities and near-exact *ab initio* references. Foundational works by Burke and coauthors on density-corrected DFT found that evaluating DFAs on the Hartree–Fock (HF) density without self-consistency yields more accurate energies for important prototypical examples including e.g., stretched heteronuclear bonds or torsional barriers in flexible molecules.^4–6^ Hence, accurate models for electron densities would benefit the developments of next-generation DFAs as well as machine-learned surrogate models for force fields^7–9^ and prediction of other properties of atomistic systems^10–12^ throughout physics, chemistry, and biology.

Large, polarizable systems require accurate treatment of nonlocal electronic correlations, such as van der Waals (vdW) dispersion, which affects both energies and ρ­(**r**) (and related properties). Nevertheless, most of the popular vdW methods, such as the DFT-D family by Grimme et al.^13–15^ or exchange-hole dipole moment (XDM) model of Johnson and co-workers,^16–18^ treat vdW dispersion as *a posteriori* energy correction without accounting for the resulting vdW polarization of ρ­(**r**). While this effect may be negligible in small molecules, it becomes visible in large, polarizable systems, where dispersion-driven stabilization is significant.^19,20^ Recent studies suggest that vdW interactions can induce substantial polarization in ρ­(**r**) for complex systems, e.g., π-stacked supramolecular dimers or molecules on a surface,^20–24^ but a comprehensive assessment of the relative role of vdW-induced ρ­(**r**) polarization in comparison to semilocal (or hybrid) DFAs remains lacking.

Beyond being an experimental observable and a basic input to DFAs, ρ­(**r**) serves as a descriptor for various chemical analyses, including molecular electrostatic potential (ESP) maps,^25^ electron localization functions,^26,27^ the quantum theory of atoms in molecules (QTAIM),^28,29^ and noncovalent interactions (NCI) visualization.^30,31^ While tools like ESP maps or NCI isosurfaces have become standard in interpreting intermolecular interactions, they often rely on density from semilocal DFAs or even atom-additive promolecular density—both of which ignore vdW-induced polarization.^30,32^ As a result, dispersion interactions are only partially captured,^32,33^ and the corresponding interaction regions may appear fragmented and incomplete.

Here, we propose an efficient approach to include vdW dispersion effects into ρ­(**r**) obtained from a (semilocal) DFA. This is achieved by using the many-body dispersion (MBD)^34^ model, which has been successfully applied to describe anisotropic molecular polarizabilities,^35–38^ polarization responses to external electric fields,^39–41^ optical excitonic spectra,^42^ and to accurately capture the effects of polarization and vdW dispersion in (bio)­molecules and materials.^20,22,24,43–46^

We employ the newly developed fully coupled and optimally tuned variant, MBD@FCO, and validate this method by benchmarking against accurate *ab initio* calculations. Applying it to the S12L^47^ and L7 ^48^ data sets of large organic dimers, we show that vdW interactions can displace electron density by up to 80% of the DFA-induced shift—or even exceed it, as observed in alkane chains. We further reveal that vdW polarization effects scale linearly with system size in polyaromatic hydrocarbons, highlighting their growing relevance in extended systems. Notably, MBD-induced density shifts contribute up to 4 kcal/mol to electrostatic potential in π-stacked complexes and proteins, suggesting a significant role in modulating long-range electrostatic interactions. Incorporating vdW-induced polarization also leads to striking changes in NCI isosurfaces, yielding smoother and more connected interaction regions. These findings suggest that dispersion interactions leave a measurable fingerprint in the electron density, with implications for both qualitative interpretation and quantitative energetics of noncovalent interactions. By revealing these subtle yet consequential effects, our study bridges the gap between dispersion energy models and density-based chemical analysis, and opens the door for a more unified treatment of noncovalent interactions in DFT.

## Results

### Fully-Coupled and Optimally-Tuned Many-Body Dispersion Method

The MBD method efficiently captures many-body polarization and dispersion effects to infinite order in perturbation theory^49^ using coupled quantum Drude oscillators (QDOs) to model collective electron density fluctuations. Each oscillator, effectively representing the response of valence electrons of an atom, is defined by three parameters: mass *m*, frequency ω, and charge *q*. To map atoms onto oscillators, we employ the optimized QDO parametrization (vdW-OQDO), designed to accurately describe atomic response to electric fields^41^ and vdW binding curves of atomic dimers.^50^ This allows us to determine {*m*, ω, *q*} solely from two response properties of atoms: the static polarizability (α_0_) and the dipolar dispersion coefficient (*C*_6_)—both obtained from reliable free-atom reference data and semilocal electron density.^51^

Interoscillator coupling in MBD is introduced using the dipole–dipole interaction tensor **T**_*AB*_. The widely used MBD@rsSCS variant^49^ employs empirical short-range damping in **T**_*AB*_, tuned for coupling with a specific DFA. This DFA+MBD@rsSCS approach is tailored to accurately reproduce total binding energies of dimers,^49^ but the pure MBD@rsSCS dispersion energy is not comparable to *ab initio* reference (Figure 1). Such an issue is inherent for any dispersion correction method relying on empirical DFA-dependent damping functions,^53–56^ as illustrated also for the XDM method in Figure 1. Instead, here we use the fully coupled dipole interaction (without DFA-specific short-range damping, see eq 9) and the vdW-OQDO parametrization, providing both accurate dispersion energies and vdW-induced density polarization. This parameter-free MBD variant, namely MBD@FCO, is applied throughout.

**Figure 1:**
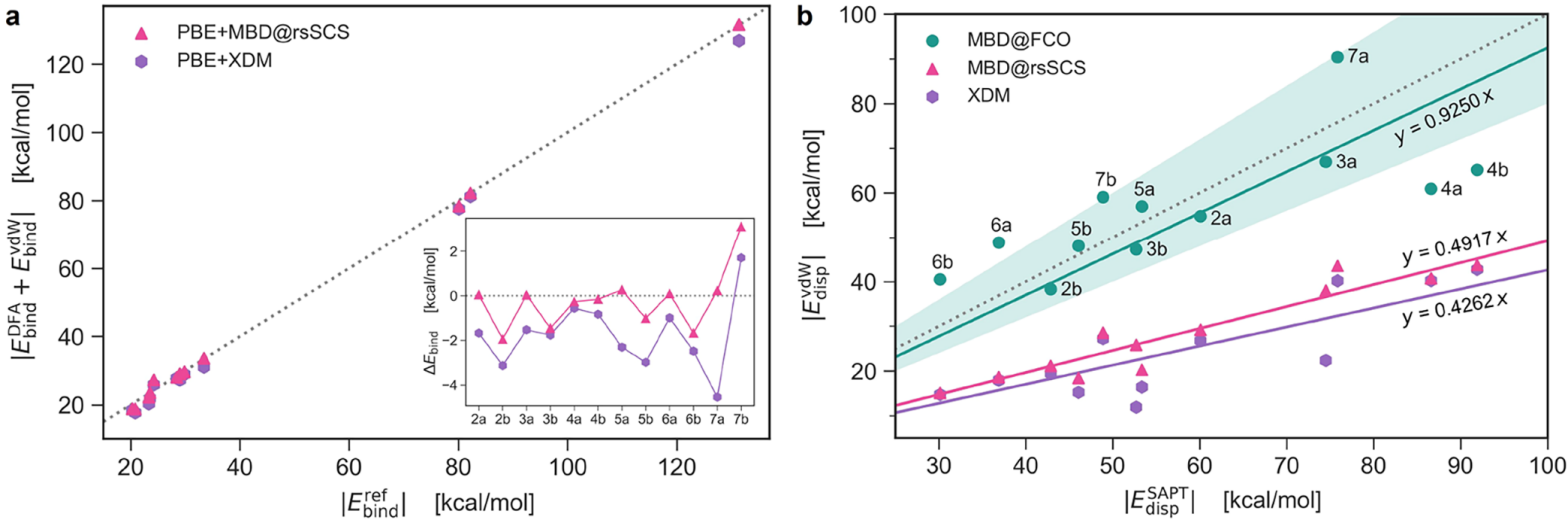
MBD@FCO method predicts accurate dispersion energies. (a) Parity plot of the total DFA+vdW binding energy magnitude from PBE+MBD@rsSCS and PBE+XDM methods versus the reference experimental binding energies for the S12L data set.^47^ The gray dotted line corresponds to the perfect correlation, while the inset shows the error Δ*E* = *E*_DFA+vdW_ – *E*_ref_ resolved per system. (b) Parity plot of the dispersion energy magnitudes *E*_disp_^(2)^ as computed by SAPT-DFT^52^ versus *E*_vdW_ from the MBD@FCO, MBD@rsSCS and XDM methods for the S12L data set. The solid lines display linear fits of data points, and the gray dotted line marks the perfect correlation, with the shaded area highlighting ±20% interval around.

In Figure 1b, we show the dispersion interaction energies for the S12L data set of supramolecular dimers,^47^ excluding the DFA contribution. These results, for two MBD variants and XDM, are compared to the benchmark values^52^ from the density-functional based symmetry-adapted perturbation theory (SAPT-DFT).^57,58^ The MBD@FCO variant yields dispersion energies closely scattered around the perfect correlation line, while MBD@rsSCS and XDM systematically underestimate dispersion by about 50%. A similar observation was reported in ref 56 (see their Figure 8), where the SAPT+MBD scheme—with a lightly damped MBD variant designed to remain valid down to short intermolecular distances—was also shown to reproduce SAPT2+(3) dispersion energies accurately. This further supports our conclusion that physics-based damping yields dispersion energies consistent with benchmark references, whereas DFA-fitted damping functions can obscure physical interpretation.

The remaining discrepancy between MBD@FCO and SAPT (MARE = 18%) is primarily due to the neglect of beyond-dipole couplings in the MBD model, which can contribute up to 30% of the total dispersion energy at equilibrium.^38,59^ We remark that for large systems, such as the S12L data set, where long-range interactions prevail, MBD@FCO performs more accurately than for small molecular dimers from the S66 data set (see Figure S1 in the Supporting Information), where the accurate description of short-range interactions and damping of dispersion interactions are relatively more important.

Ambrosetti et al.^42^ further demonstrated that the fully coupled MBD model can semiquantitatively reproduce molecular optical excitation spectra. This ensures that the fully coupled MBD method delivers physically sound description of collective quasi-plasmonic fluctuation modes, interplay of which creates a vdW-induced polarization of electronic density. In the following, we show that beyond the accurate dispersion energies, the MBD@FCO method yields physically relevant vdW-induced density polarization.

### MBD Predicts vdW-Induced Density Polarization

Collective charge density fluctuations, which mediate vdW dispersion interaction, induce density polarization.^20,21,23^ Within the MBD framework, vdW-induced density polarization is naturally defined as the difference between the charge densities of interacting and noninteracting QDOs, constituting the model^20,60^

$$\rho_{\mathrm{pol}}\left(r\right)=\left\langle \Psi \right|\mathrm{\rho ̂}\left|\Psi \right\rangle -\left\langle \Psi_{0}\right|\mathrm{\rho ̂}\left|\Psi_{0}\right\rangle ,\;\mathrm{\rho ̂}=\overset{N}{\underset{A=1}{\sum }}q_{A}\delta \left(r-{\mathrm{r̂}}_{A}\right)$$ 1

where Ψ­({**r**_*A*_}) and Ψ_0_({**r**_*A*_}) are Gaussian ground-state wave functions of interacting and noninteracting oscillators, respectively. For the explicit ρ_pol_(**r**) expression, please see the Supporting Information Section S2.

The optimal choice of QDO charges *q*_*A*_ is essential for accurate density predictions. Using the vdW-OQDO parametrization, we assign species-specific charges based on polarizabilities and *C*_6_ coefficients, in contrast to earlier methods using arbitrary *q*_*A*_ = 1 au charge values^20,24^ (see Figure S7 in the Supporting Information).

To focus on the interaction-induced polarization, we compute the density change Δ*ρ*_pol_(**r**) for a dimer (D) composed of two monomers (M1, M2)

2 $$\Delta \rho_{\mathrm{pol}}\left(r\right)=\rho_{\mathrm{pol}}^{D}\left(r\right)-\rho_{\mathrm{pol}}^{M1}\left(r\right)-\rho_{\mathrm{pol}}^{M2}\left(r\right)$$

We also calculate the vdW polarization of density using the self-consistent Tkatchenko–Scheffler method (sc-TS)^21,51^ on top of the Perdew–Burke–Ernzerhof (PBE) functional^61^

3 $$\Delta \rho_{\mathrm{sc}‐\mathrm{TS}}\left(r\right)=\left[\rho_{\mathrm{PBE}+\mathrm{TS}}^{D}\left(r\right)-\rho_{\mathrm{PBE}+\mathrm{TS}}^{M1}\left(r\right)-\rho_{\mathrm{PBE}+\mathrm{TS}}^{M2}\left(r\right)\right]-\left[\rho_{\mathrm{PBE}}^{D}\left(r\right)-\rho_{\mathrm{PBE}}^{M1}\left(r\right)-\rho_{\mathrm{PBE}}^{M2}\left(r\right)\right]$$

to analyze the effects of self-consistency at the DFT level.

We benchmark MBD@FCO and sc-TS on small dispersion-bound hydrocarbons against the double density difference between coupled-cluster singles and doubles (CCSD) and Hartree–Fock (HF) levels of theory, Δρ_CCSD–HF_ (**r**), which accounts for the contribution of (long-range) electronic correlations to the density:

4 $$\Delta \rho_{\mathrm{CCSD}‐\mathrm{HF}}\left(r\right)=\left[\rho_{\mathrm{CCSD}}^{D}\left(r\right)-\rho_{\mathrm{CCSD}}^{M1}\left(r\right)-\rho_{\mathrm{CCSD}}^{M2}\left(r\right)\right]-\left[\rho_{\mathrm{HF}}^{D}\left(r\right)-\rho_{\mathrm{HF}}^{M1}\left(r\right)-\rho_{\mathrm{HF}}^{M2}\left(r\right)\right]$$

CCSD produces highly accurate densities for small molecules, as shown by Mezei et al.^63^ by benchmarking against the CCSDTQ reference. We found that in dispersion-bound dimers at large distances, CCSD–HF difference provides an excellent reference for the density distortion due to dispersion, with neglected triples having only a minor effect on densities (see Figure S9 of the Supporting Information). Since MBD@FCO captures only the dispersion-induced density contribution, we restrict our benchmarks to dispersion-dominated dimers, where Δρ_CCSD–HF_ can be meaningfully compared to MBD density shifts. For mixed or electrostatics-dominated systems, Δρ_CCSD–HF_ contains substantial nondispersion contributions, making direct comparison with MBD inappropriate. A rigorous assessment in such cases would require dispersion-only density components from wave function methods such as SAPT, which are not yet available.

The choice of the mean-field method to subtract from correlated densities is generally nontrivial and also affects the computed Δρ. In this work we use the Hartree–Fock density as a baseline, since it contains no electron correlation and therefore no dispersion. The resulting difference Δρ_CCSD–HF_ provides a clean measure of correlation-induced density distortions in dispersion-dominated systems, as supported also by good agreement between CCSD–HF and SAPT2­(+3) interaction energies (Figure S11 of the Supporting Information). Alternative mean-field references, such as PBE, already include approximate correlation effects that obscure a straightforward isolation of dispersion, and moreover suffer from delocalization errors (see Section S3 of the Supporting Information). For these reasons, we regard CCSD–HF as the most reliable practical reference, while acknowledging its limitations.

Plane-averaged density changes Δρ­(*z*) = ∫∫Δρ­(**r**)­d*x*d*y* as a function of the *z*-coordinate along the dimer axis are shown for methane and pentane dimers in Figure 2, with results for other four small dimers available in Figure S2 of the Supporting Information. The CCSD–HF, sc-TS and MBD@FCO methods qualitatively agree in predicting charge redistribution due to vdW dispersion interactions, aligning with their renowned Feynman’s picture: net charge accumulation between monomers causes an electrostatic forces between this distorted electronic cloud and the nuclei of a given molecule, resulting in effective intermolecular attraction.^64^ MBD@FCO accurately describes density polarization between the monomers, while discrepancies near the nuclei likely stem from the harmonic potential approximation in MBD, which differs from the Coulomb potential in real systems. The self-consistent sc-TS method agrees more closely with CCSD in the density tails, suggesting the importance of self-consistency, which is the focus of ongoing work on MBD@FCO.

**Figure 2:**
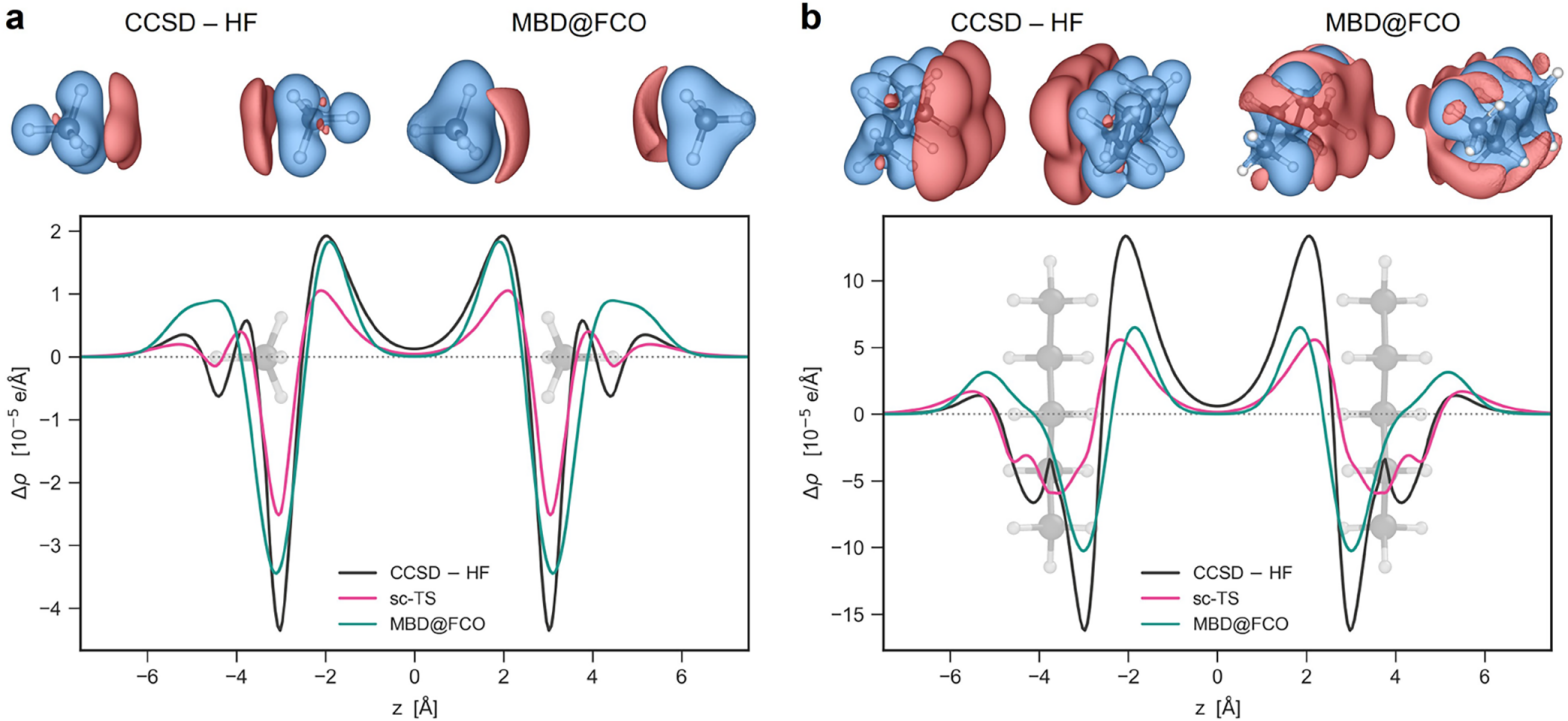
Benchmarking densities against coupled-cluster calculations. vdW-induced charge density polarization in (a) methane and (b) pentane dimers computed by CCSD–HF, sc-TS and MBD@FCO methods. The plots show plane-averaged density polarization Δρ­(*z*) with the monomer geometries represented along the distance axis. The isosurfaces (top) illustrate Δρ­(**r**) in the 3D space as calculated by CCSD*-*HF and MBD@FCO methods with the isovalues of 1.5 × 10^–6^ and 2 × 10^–6^ e/Å^3^ for methane and pentane dimers, respectively. The red and blue colors denote accumulation and depletion of charge density, correspondingly. The isosurface plots were produced using the VESTA software.^62^

The isosurfaces of Δρ­(**r**) displayed in Figure 2 witness that Δρ_pol_(**r**) from MBD@FCO captures the key features of Δρ_CCSD–HF_(**r**) redistribution also in 3D space. The remaining differences outside monomers can be attributed to the dipole coupling approximation used in the MBD model. We note that dipole–quadrupole couplings, which are known to be important for reproducing dispersion dipoles,^65^ can be incorporated in a perturbative fashion, using the MBD dipole-coupled ground state as the starting point.

As the quantitative measure, we compute the integral of density displacement over charge-accumulating regions (cf. red-colored densities in Figure 2, top), defining *Q*_vdW_ = ∫_Ω_Δρ­(**r**)­d^3^ *r*, where Ω is the region with Δρ_pol_(**r**) > 0. We arbitrarily specify Ω as charge-accumulating regions (charge-depleted regions can be equivalently chosen), since integral of Δρ over all space is zero by construction. Thus, *Q*_vdW_ captures the total charge displaced due to vdW interactions and serves as a scalar measure of vdW polarization strength. We found that MBD@FCO well reproduces *Q*_vdW_ from CCSD–HF, with a MARE of 24% (see Figure S3 in the Supporting Information), which can serve as the accuracy estimate for our method. Having validated MBD@FCO against the high-level reference, we further apply it to large noncovalent complexes to study the scaling of vdW density polarization with system size.

### Dispersion-Induced Density Polarization Grows with System Size

To assess the scaling of vdW density polarization with system size, we investigate 24 organic complexes ranging from 24 to 240 atoms. These include the 12 host–guest dimers from the S12L data set,^47^ and 11 systems forming the extended L7+ data set: the original L7 set,^48^ C_60_ dimer, circumcoronene (C3C3PD), circum–circumcoronene (C4C4PD), and a DNA-ellipticine complex.^66^ A parallel-displaced benzene dimer serves as a reference in both data sets. Atomic geometries and nomenclature follow original sources; additional polyaromatic hydrocarbons (PAHs) geometries were optimized using PBE+MBD@rsSCS in FHI-aims with “tight” settings (see Supporting Information Section S1.4).

The vdW-induced charge displacements, Δρ_pol_(**r**), were computed for all systems using the sc-TS and MBD methods, as described above, with the PBE functional as baseline. PBE was chosen for its widespread use and low computational cost, and benchmark tests with HF yield similar trends, confirming that our key observations are not method-dependent (see Supporting Information Section S1.3).

Figure 3 summarizes the results, displaying Δρ_pol_(**r**) isosurfaces from MBD@FCO. In large, polarizable systems, the displaced density concentrates in the intermonomer region and grows markedly with size. The total displaced charge, *Q*_vdW_, ranges from 0.1 to 0.3*e* (Figure 4) and far exceeds 0.01*e* found in benzene dimer. The pairwise sc-TS method yields significantly smaller *Q*_vdW_, likely due to a strong damping at shorter distances.

**Figure 3:**
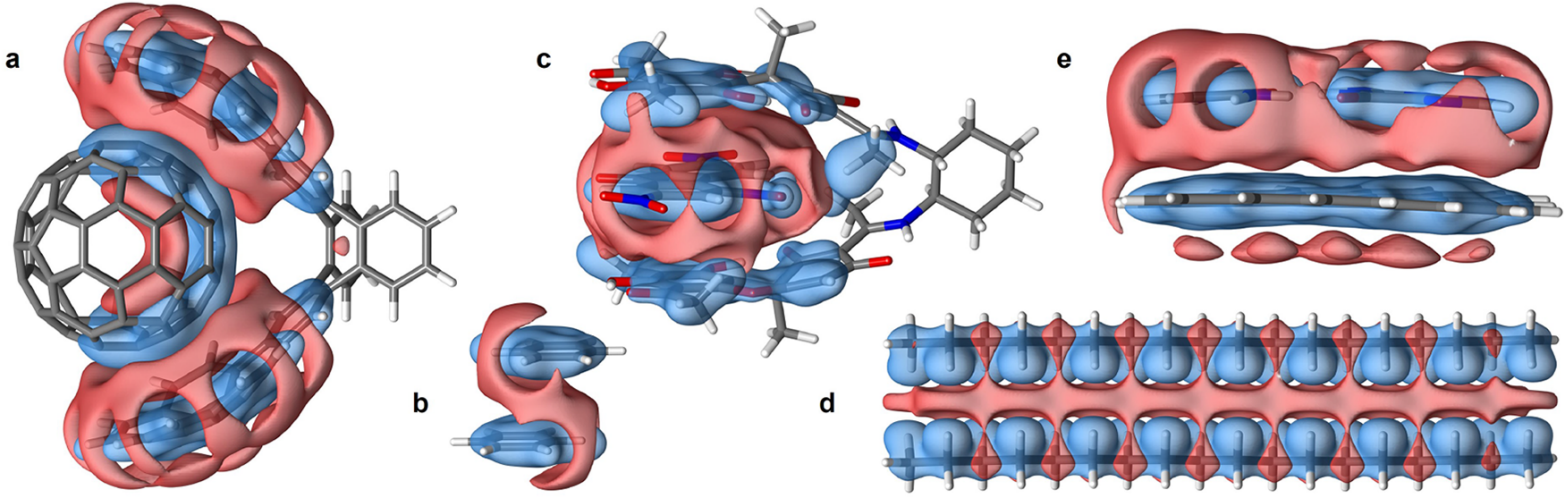
vdW-induced charge density polarization in S12L and L7+ data sets. Isosurfaces of vdW density polarization between a complex and its isolated monomers for selected systems from S12L and L7+ data sets (with isovalue 3 × 10^–5^ au, unless otherwise noted): (a) C_60_ buckyball catcher (4a, S12L); (b) benzene dimer (1, S12L, 2 × 10^–5^ au); (c) TNF “pincer” complex (3a, S12L, 6 × 10^–5^ au); (d) octadecane dimer (CBH, L7+); (e) guanine-cytozine dimer on circumcoronene (C3GC, L7+). The red and blue colors denote accumulation and depletion of charge density, respectively.

**Figure 4:**
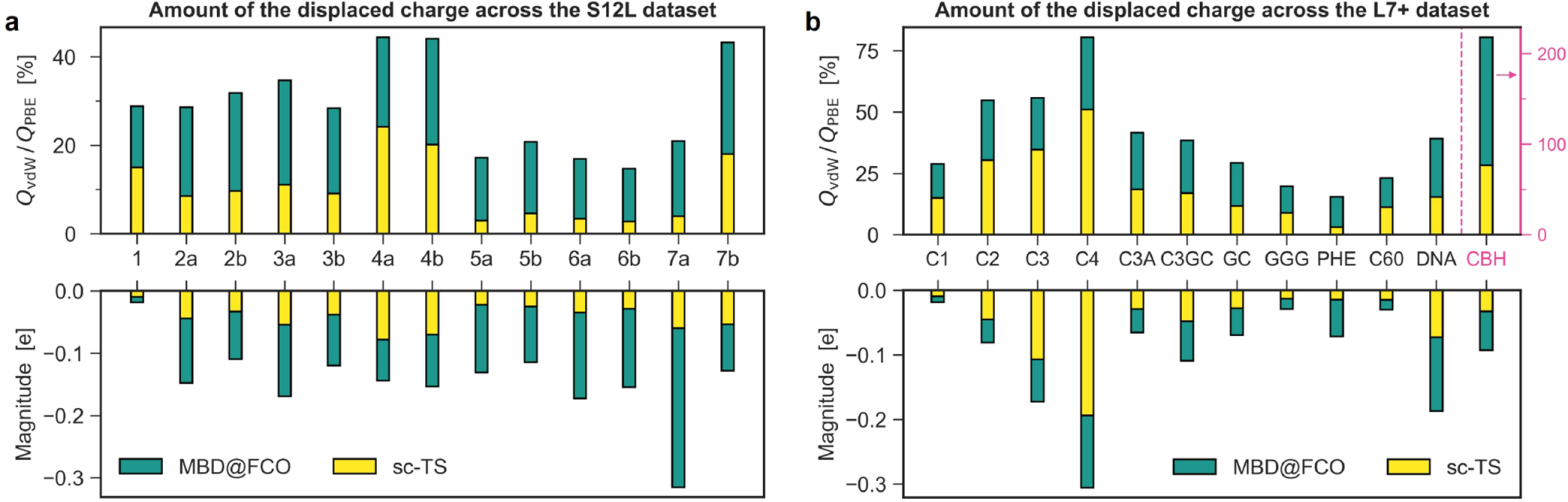
vdW-displaced charges across S12L and L7+ data sets. The total vdW-displaced charge −*Q*_vdW_ (bottom) and its ratio to the PBE-displaced charge *Q*_PBE_ (top) for (a) S12L and (b) L7+ data sets as computed by sc-TS and MBD@FCO methods. Note the separate *y*-axis for CBH in panel (b).

To contextualize these numbers, we compare *Q*_vdW_ to the analogous *Q*_PBE_, calculated using the bare PBE functional without vdW corrections. *Q*_PBE_ estimates induction effects at the PBE level, and hence the ratio *Q*_vdW_/*Q*_PBE_ reflects the relative importance of dispersion versus induction. As shown in Figure 4, this ratio reaches up to 80% in π-stacked complexes and exceeds 100% in dispersion-bound linear alkanes (CBH), demonstrating that dispersion can dominate over DFA induction. Notably, this ratio reaches 76% even at the sc-TS level, while MBD further increases vdW charge polarization by enabling delocalized collective density fluctuations along the chain (see Figure 4b).

We remark than in the CBH chain, induction effects might be suppressed due to a high symmetry. To test this, we examined a diacylglycerol dimer—a simplified model of a lipid—composed of long alkyl chains but having a lower symmetry (see Section S1.5 of the Supporting Information). There, *Q*_vdW_/*Q*_PBE_ drops to 53%—lower than in CBH, yet still a substantial contribution. We estimate the accuracy of *Q*_vdW_ from MBD@FCO to be about 20%, based on its performance for dispersion energies (Figure 1b) and linear correlation between *Q*_vdW_ and MBD energies (see Figure S3).

To explore scaling behavior, we analyze stacked PAH dimers from benzene (C1C1PD) to circum–circumcoronene (C4C4PD). As shown in Figure 5a, *Q*_vdW_ grows linearly with system size. This linear trend is supported by CCSD–HF calculations on benzene through coronene dimers (see details in Supporting Information). The difference in slope between CCSD–HF and MBD@FCO likely originates from beyond-dipole dispersion and induction differences between CCSD and HF in aromatic systems (see Figure S9b).

**Figure 5:**
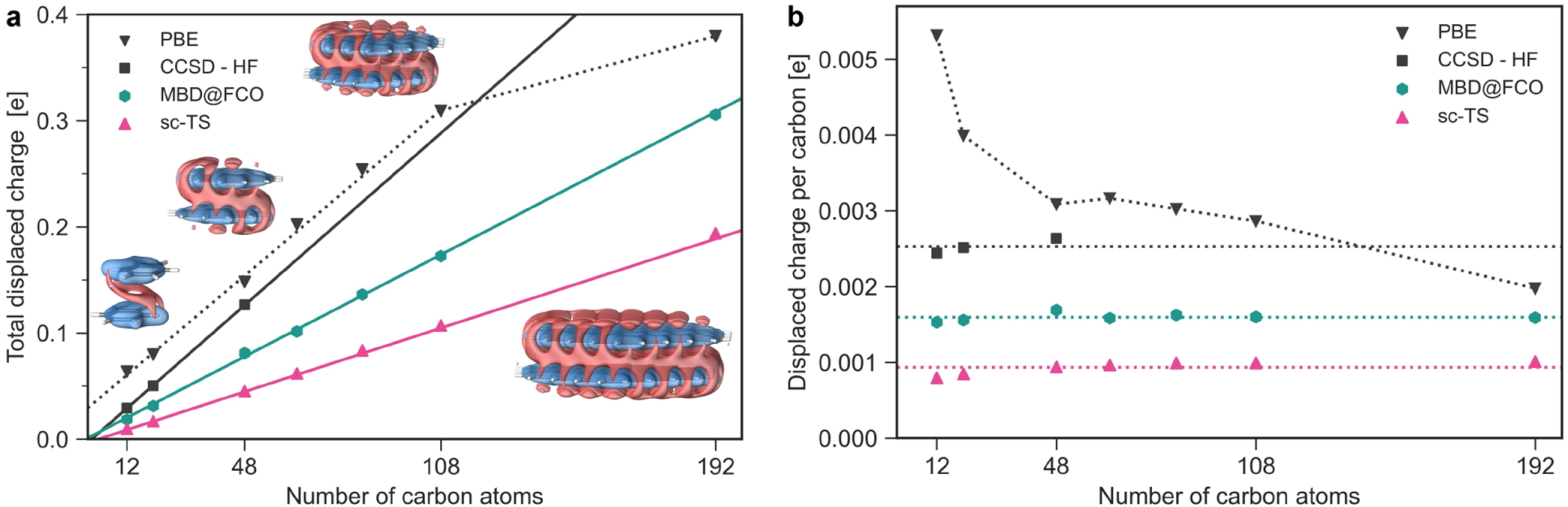
Linear scaling of vdW polarization with system size in polyaromatic hydrocarbons. (a) The vdW-displaced charge as computed by CCSD–HF, MBD@FCO and sc-TS plotted against the number of carbon atoms in PAH dimers. Linear fits are shown to guide an eye, and the PBE-displaced charge is displayed as a measure of induction effects. The insets display the 3 × 10^–5^ au isosurfaces of the Δρ_pol_(**r**) for the selected PAHs. (b) *Q*_vdW_ normalized per carbon atom as a function of size. Horizontal lines mark the mean values, while data points for PBE are connected to guide an eye.

Interestingly, *Q*_PBE_ shows signs of saturation in the largest PAH, while *Q*_vdW_ continues to grow—with their ratio reaching 80%. Such a behavior can be better understood when normalizing a displaced charge per carbon atom, as shown in Figure 5b. While normalized *Q*_vdW_ remains constant for both MBD@FCO and sc-TS, *Q*_PBE_ shows a marked decline. Similar trend is observed across other functionals (see Figure S4b in the Supporting Information), suggesting that induction effects fall with increasing size. This reflects the diminishing edge effects and increasing bulk-like (highly symmetric) character as hydrocarbon flakes grow—reducing induction but not dispersion. On the contrary, the constant normalized *Q*_vdW_ highlights the increasing dominance of dispersion effects over induction in extended π-systems.

Altogether, these findings reveal that vdW dispersion not only contributes significantly to energy stabilization, but also induces pronounced and size-dependent charge polarization effects. In the next section, we explore how these density shifts reshape the electrostatic potential, revealing implications for noncovalent interactions in chemistry and biology.

### Electrostatic Potential and Energetic Impacts of MBD Density Polarization

To measure the impact of MBD@FCO density polarization on interaction energies, we investigate how it affects the electrostatic potential (ESP)—a local observable that depends sensitively on the entire electron density distribution through the Coulomb kernel. The ESP describes the first-order interaction energy between a molecule and a unit test charge placed at a given point in space. We refer the reader to Section S2.4 of the Supporting Information for the derivation of ESP within the MBD framework.

Mapping the ESP onto the vdW surface of a molecule provides intuitive insight into how it might interact with other molecules or charged species via electrostatic forces. Although such maps reflect only electrostatic contributions, they remain widely used in chemistry and biomolecular modeling due to their predictive utility.

While in previous sections we focused on density differences between a dimer and constituting monomers, to evaluate the ESP we use only ρ_pol_ of a dimer. This approach provides a robust approximation to the long-range part of the vdW-induced ESP shift, as validated via benchmarking against the CCSD–HF reference (Figure S8). We remark that at distances shorter than 3 Å, MBD@FCO might overestimate ESP, as its soft harmonic potential is parametrized to match the long-range atomic response properties.

Figure 6 illustrates the influence of MBD-induced density polarization on ESP using the C_70_ catcher (molecule 4b from S12L data set) as an example. The polarization predominantly affects the tails of the electron density (Figure 6a and inset), leading to a substantial positive shift in the electrostatic potential. At typical vdW contact distances (3–4 Å), the electrostatic interaction between a unit test charge and the MBD@FCO density reaches 4 kcal/mol. This significantly alters the decay of the total PBE+MBD@FCO electrostatic potential compared to the PBE baseline (Figure 6b). Corresponding ESP maps (Figure 6c,d) confirm that these shifts are most pronounced near the π-conjugated carbon rings, suggesting that neglecting vdW-induced density polarization in such regions may lead to substantial errors—up to 4 kcal/mol—in charge-density interaction energies. Interestingly, the MBD@FCO contribution has an opposite sign compared to the PBE distribution. This can be attributed to the different behavior of PBE and MBD@FCO densities, as shown in Figure 6a. Despite being at the level of 10^–3^ au, the vdW polarization of density contributes to ESP comparably to PBE at longer range, underscoring its practical importance in polarizable environments.

**Figure 6:**
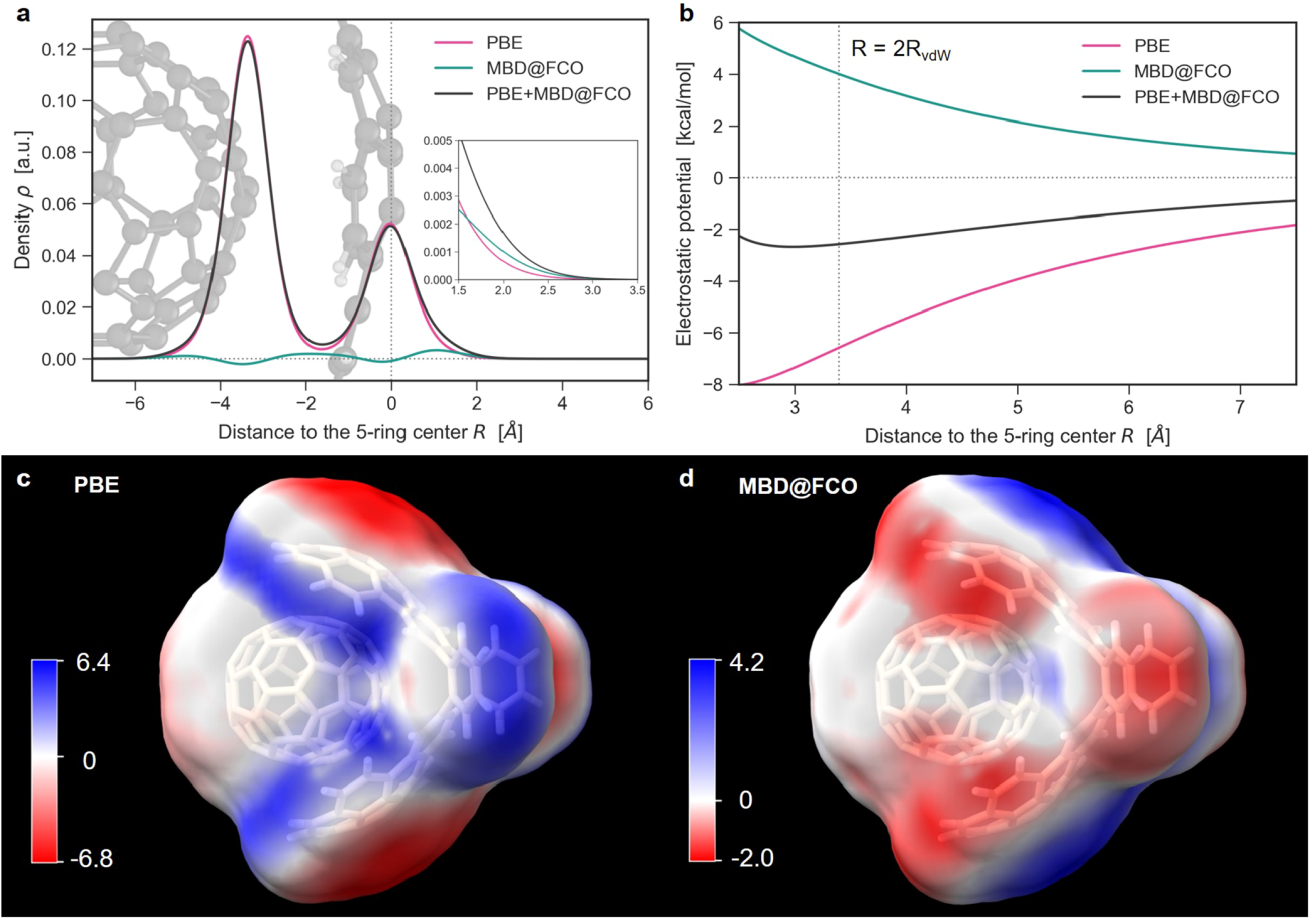
Electrostatic potential of C_70_ catcher modified by the MBD density polarization. (a) Density profile along the normal to the catcher’s “hand” passing through its geometric center (the atomic structure superimposed to scale). The inset shows the decay of the density tails at an enlarged scale. (b) ESP decay along the same line for the three methods. (c, d) ESP map (in kcal/mol) on the 2*R*_vdW_ surface as computed using (c) PBE and (d) MBD@FCO densities. The images are created using the ChimeraX software.^67^

We note that changes in ESP are often misinterpreted as reflecting purely local variations of the electron density. However, as Wheeler and co-workers have shown,^68,69^ such a local viewpoint can be misleading. Our own results provide complementary evidence: although MBD density polarization increases the electron density above the aromatic ring (Figure 6a), the corresponding ESP shift in this region is positive (Figure 6d). This reinforces the inherently nonlocal nature of the ESP and underscores the need for caution in attributing ESP features to local density changes.

We next extend this analysis to proteins, where vdW polarization effects exhibit pronounced chemical selectivity. As a representative example, we analyze the Fip35-WW domain, a prototype protein whose collective dispersion interactions with solvent have been previously characterized by Stöhr and Tkatchenko.^46^ From the folding trajectory obtained by Shaw et al.^70^ using classical force fields, we selected three representative atomic structures corresponding to the unfolded, transition, and folded states. To isolate intraprotein effects and enable tractable DFT calculations, we removed water molecules, counterions, and the net protein charge, producing neutral gas-phase geometries for both PBE and MBD@FCO calculations of electron density and ESP.

Figure 7a compares ESP maps for the unfolded state derived from PBE and MBD@FCO densities. While PBE yields overall stronger ESP magnitudes, MBD-induced contributions are significant, reaching 3.5 kcal/mol. In certain local regions, the ESP from MBD@FCO even exceeds that from PBE. To quantify this, we identify seven amino acid residues where the MBD@FCO ESP is maximal. Figure 7b compares these values with the corresponding PBE ESP evaluated in the same regions. While MBD@FCO contributes significantly less than PBE near Lys3 and Pro6 residues, it exceeds PBE close to the benzene-like Trp8 residue. Near Pro5, Tyr20, His23 and Phe30 aromatic residues, the MBD@FCO also contributes remarkably to the electrostatic potential, amounting between 25 and 50% relative to the PBE potential. This trend reinforces our findings from the C_70_ catcher and highlights that vdW density polarization is highly dependent on chemical composition, being particularly pronounced in π-conjugated environments—ubiquitous in realistic protein structures.

**Figure 7:**
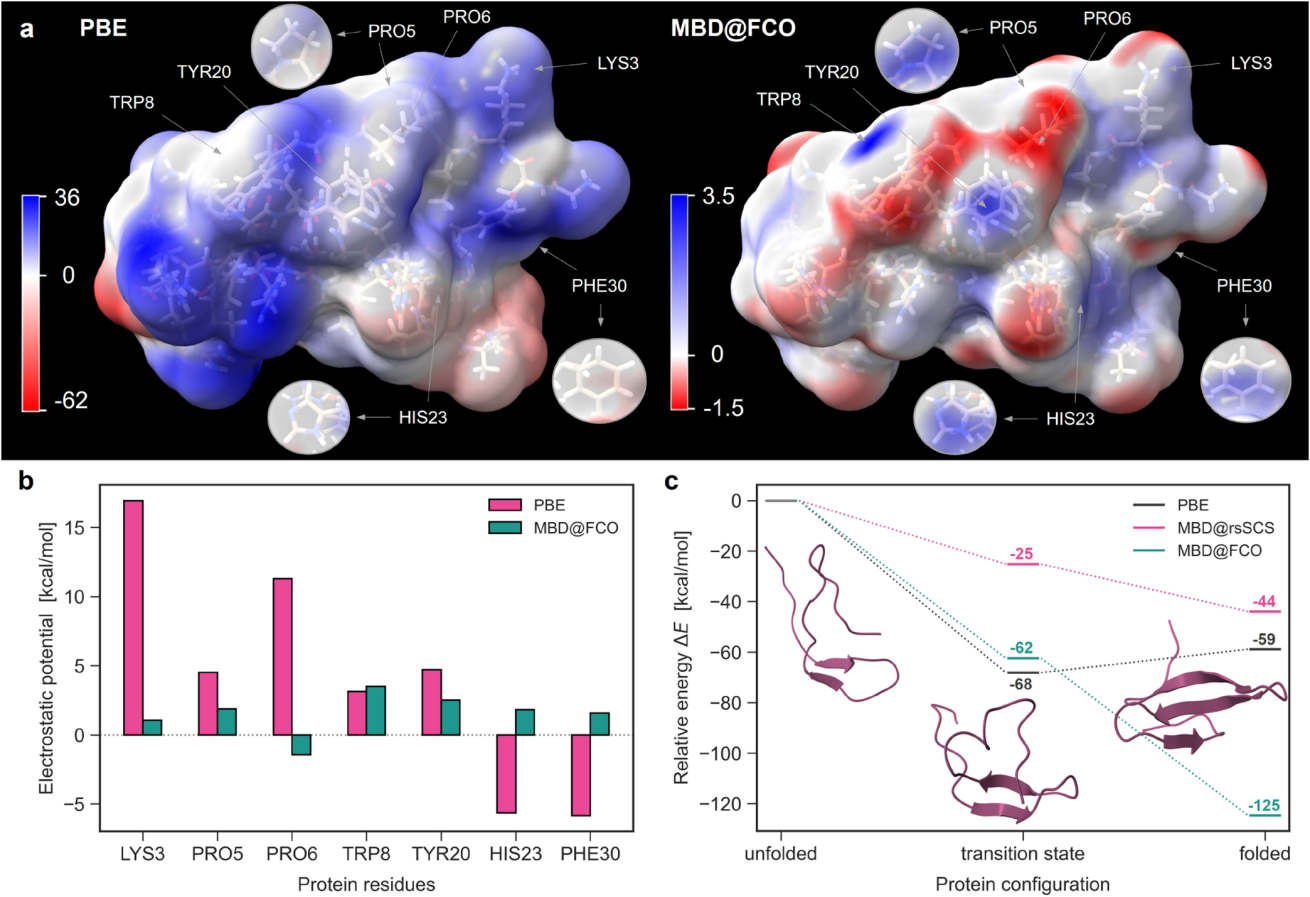
MBD density polarization selectively modifies electrostatic potential of the protein. (a) ESP map (in kcal/mol) on the solvent-accessible surface of Fip35-WW protein (in the unfolded state) computed using PBE (left) and MBD@FCO (right) densities. Residues where the MBD@FCO contribution to electrostatic potential is the most significant are indicated with arrows and in the insets. The images are created using the ChimeraX software.^67^ (b) Electrostatic potential near residues from panel (a) based on PBE and MBD@FCO densities. (c) Relative energy of Fip35-WW protein along the folding path as computed by PBE, MBD@rsSCS and MBD@FCO methods. The secondary structures of the sampled configurations are displayed.

Furthermore, experimental studies have shown that local electric fields can catalyze chemical transformations in enzyme active sites.^71^ The reported magnitude of field-induced barrier reduction (7.3 kcal/mol) is comparable to the MBD-induced electrostatic shifts observed here. This suggests that including vdW-induced density polarization near active sites may be essential for accurate theoretical modeling of enzymatic catalysis.

Beyond electrostatic potentials, vdW dispersion forces themselves play a fundamental role in determining the stability of biomolecular structures. In particular, they are a major driving force behind protein folding, alongside hydrogen bonding, hydrophobic effects, and ion pairing.^72,73^ Figure 7c shows the relative energies of the three Fip35-WW conformations along the folding path. When long-range vdW interactions are omitted (as in semilocal PBE), the transition state is incorrectly predicted to be more stable than the folded state. In contrast, both MBD@rsSCS and MBD@FCO recover the correct energetic ordering, underscoring the essential stabilizing role of dispersion. The MBD@FCO method, previously validated on large systems from the S12L benchmark (Figure 1b), predicts a stabilization of 125 kcal/mol for the folded state. This large value reflects the cumulative effect of dispersion interactions across the protein and may be further modulated when vdW-induced density polarization is included self-consistently. While these calculations neglect solvent and ionic effects, they provide compelling evidence for the critical role of vdW forces in protein folding, in line with prior literature.^46,72,73^

In the next section, we explore how vdW-induced density polarization modifies the visualization and interpretation of noncovalent interactions by analyzing its impact on reduced density gradient features in space.

### MBD-Corrected Densities Yield Smooth Non-Covalent Interaction Regions

The noncovalent interaction (NCI) analysis framework^30,31^ enables visualization of weak interactions based on the reduced density gradient (RDG)

5 $$s\left(r\right)=\frac{1}{2{\left(3\pi^{2}\right)}^{1/3}}\frac{\left|\nabla \rho \left(r\right)\right|}{\rho^{4/3}\left(r\right)}$$

which approaches zero in regions of low density and weak interactions. Plotting *s*(**r**) vs ρ­(**r**) reveals characteristic features—stripe-like patterns in (ρ,*s*) space—that serve as fingerprints for NCIs (see Figure S14). The nature of these interactions is encoded in the second eigenvalue λ_2_ of the electron-density Hessian (λ_1_ < λ_2_ < λ_3_), with λ_2_ < 0 indicating attraction, while λ_2_ > 0 signifies repulsion.^30,31^ The product ρ­(**r**) sign­(λ_2_) effectively captures interaction strength, and it is used to color RDG isosurfaces in real space, with blue for strong attraction (e.g., hydrogen bonds), green for vdW dispersion, and red for steric repulsion.

To assess the influence of vdW-induced density polarization, we construct the total electron density as a simple sum ρ_tot_(**r**) = ρ_PBE_(**r**) + ρ_pol_(**r**), where ρ_pol_ is the MBD@FCO polarization density (see Section S2.2 of the Supporting Information for detailed explanations). NCI isosurfaces based on ρ_tot_ reveal systematic changes compared to the baseline PBE density (Figure 8). Green vdW regions become thicker and more connected, red steric zones sharpen, and the spatial continuity of weak interaction regions improves—especially in extended or flexible molecular systems.

**Figure 8:**
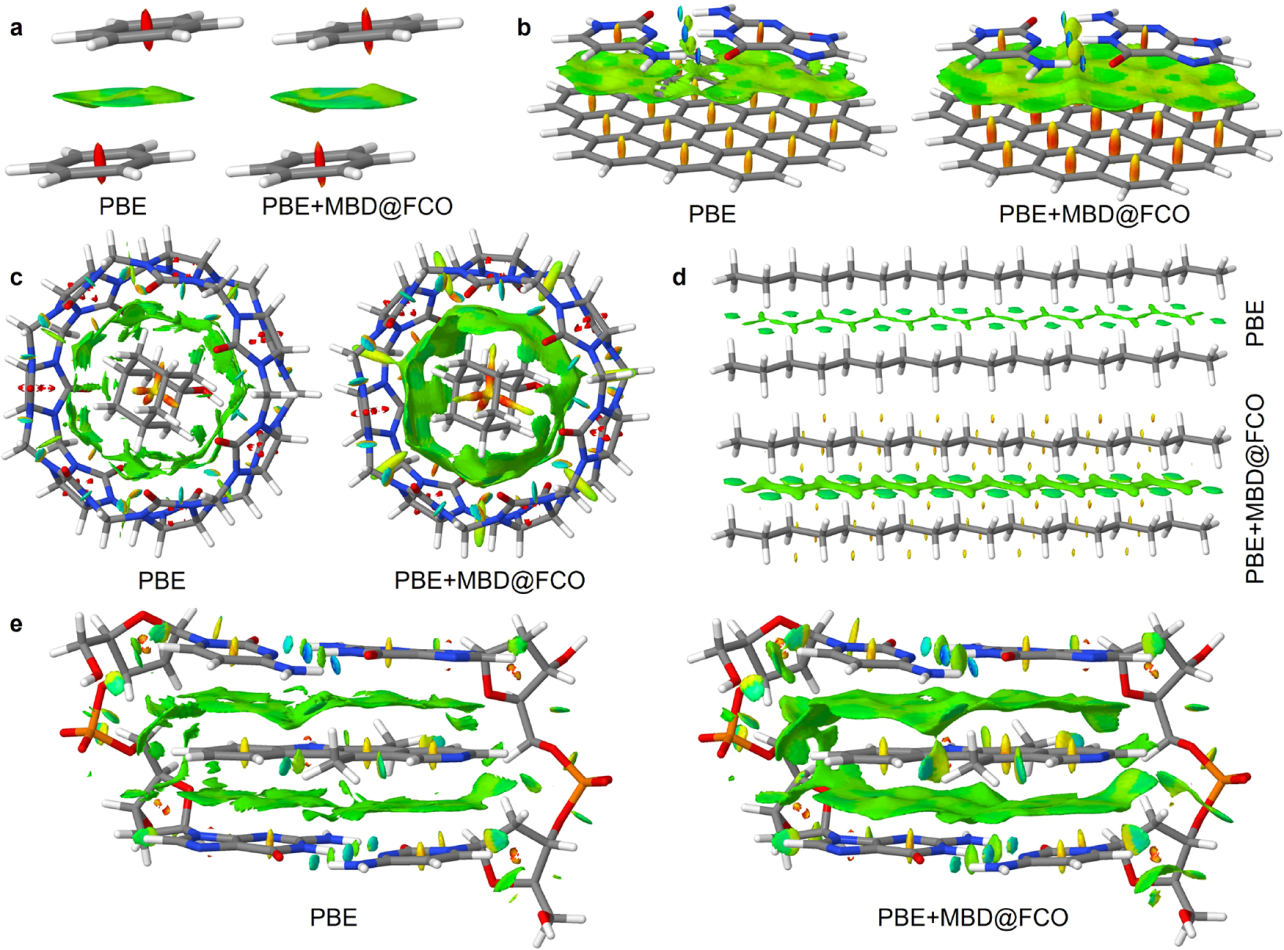
vdW contribution to the density makes NCI isosurfaces more connected. NCI isosurfaces of density gradient *s* (coloring range of ρ­(**r**) sign­(λ_2_) is given in au in square brackets) calculated using PBE and PBE+MBD charge densities for (a) benzene dimer: *s* = 0.5, [−0.02, 0.02]; (b) C3GC (L7+): *s* = 0.5, [−0.05, 0.04]; (c) 7b (S12L): *s* = 0.5, [−0.03, 0.03]; (d) octadecane dimer (CBH, L7+): *s* = 0.65, [−0.02, 0.02]; (e) DNA-ellipticine: *s* = 0.5, [−0.05, 0.05]. Isosurfaces are plotted using the Jmol software.^74^

These effects are evident in a range of systems. In the C3GC (b), 7b@S12L (c), and DNA-ellipticine (e) complexes, vdW isosurfaces become more cohesive, with clearer distinction between attractive and repulsive zones. In the octadecane dimer (CBH, Figure 8d), PBE barely captures the weak dispersion between chains, while MBD@FCO polarization reveals stronger interchain attraction and repulsive hydrogen–hydrogen contacts. Hydrogen bonds are also better resolved with inclusion of MBD@FCO, as evident in C3GC and DNA-ellipticine.

To quantify these changes, we compute the total volume of NCI isosurfaces (*V*_NCI_) using the NCImilano program.^75^ The enhancement factor γ = *V*_NCI_^PBE+MBD^/*V*_NCI_^PBE^ captures the relative growth of interaction regions upon including MBD@FCO polarization. Across S12L and L7+ benchmark systems, we observe consistent NCI volume increases, with γ in range 2–3 (Figure S14). In contrast, γ ≈ 1 for systems dominated by electrostatics or steric repulsion, highlighting the selectivity of MBD@FCO densities for dispersion-driven interactions. A full account of γ values and system-specific trends is provided in Section S5 of the Supporting Information.

The same trend is observed in biomolecules. For the folded Fip35-WW protein (Figure 9), MBD-enhanced densities yield smoother and more interconnected vdW regions, with an overall volume enhancement of γ ≈ 2.5. Local inspection around residues such as Trp8, Arg17, and Tyr20 reveals that many are encased in green “vdW cages”, indicative of additional stabilization through dispersion—consistent with Figure 7c. In contrast, PBE-derived isosurfaces appear fragmented and sparse, complicating the interpretation of interaction networks.

**Figure 9:**
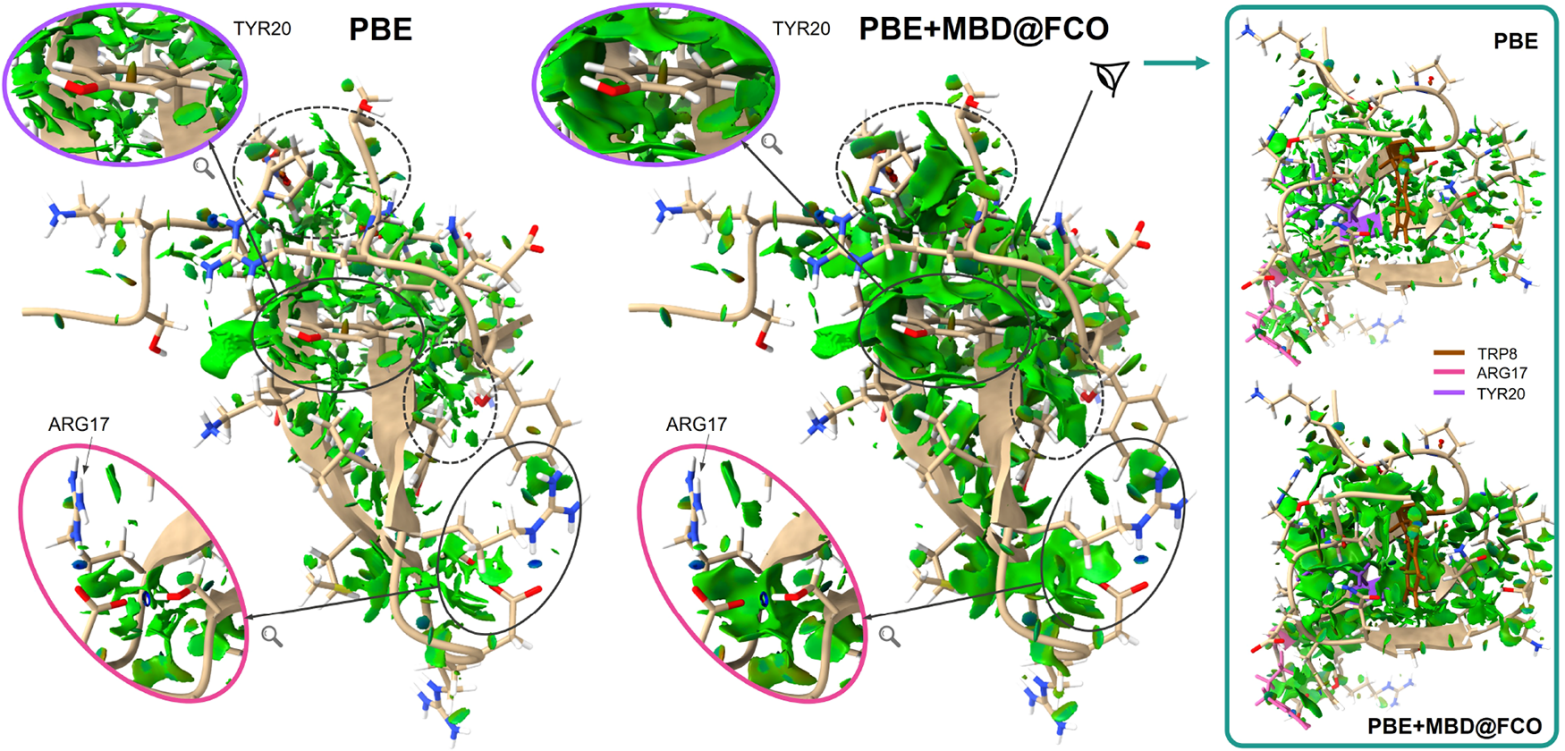
vdW-corrected density analysis highlights interacting regions in the protein. NCI isosurfaces (*s* = 0.5) of Fip35-WW protein in the folded state computed using PBE and PBE+MBD density are shown. Regions of the protein structure with the largest differences are highlighted in ellipsoids, with the vicinity of TYR20 and ARG17 residues additionally displayed in magnification. The right panel shows the view on Fip35-WW from the direction of TRP8 residue (in brown), as indicated by side eye pictogram. For reference, ARG17 and TYR20 residues shown in the central panel are colored in pink and purple, respectively. The images are created in the ChimeraX software^67^ using the density gradients calculated with the NCImilano program.^75^

NCI analysis is inherently qualitative, and its utility lies in providing intuitive visualizations of weak interactions. In our case, we observe that including MBD density polarization renders the NCI isosurfaces more coherent and connected, a feature that facilitates interpretation in large molecular systems. While such plots should not be overinterpreted, they serve as a complementary visualization to the ESP analysis, pictorially showing how dispersion-induced density shifts permeate the molecular scaffold. Moreover, integrals of the electron density over NCI regions have been shown to correlate with interaction energies.^76,77^ This indicates that NCI plots, despite their qualitative nature, can provide useful bridges between density features, interaction energetics, and visualization in complex systems.

## Discussion and Outlook

In this work, we have investigated how long-range vdW interactions, treated within the many-body dispersion framework, induce non-negligible shifts in the electron density of molecular systems. Using the MBD@FCO method, we showed that while such effects are minimal in small molecules, they become increasingly significant in larger, more polarizable systems.

These vdW-induced density deformations have tangible consequences. In the context of NCI analysis, they give rise to smoother and more physically connected isosurfaces, enhancing our ability to interpret interaction regions in large and complex systems. For example, the folded Fip35-WW protein exhibits substantial vdW-driven electrostatic shifts and clearly defined dispersion “cages” between aromatic residues. These observations underscore the central role of dispersion-induced density polarization in understanding biomolecular structure, stability, and function.

Beyond chemical analysis, our results have broader implications for modeling interactions in biological environments. Electrostatic interactions are central to the structure, dynamics, and function of proteins, which typically operate in charged environments with counterions and solvent at physiological conditions. Mechanisms such as salt bridges, cation-π interactions, and localized electric fields are known to govern protein folding, ligand binding, and catalysis. Our analysis of ESP reveals that vdW-induced density polarization can locally modify it by 3–4 kcal/mol, especially near π-conjugated residues in proteins. In some regions, the MBD-polarized density even yields stronger ESP contributions than the underlying semilocal functional, emphasizing that dispersion is not merely a secondary correction but an active contributor to electrostatic landscapes. These shifts are comparable in magnitude to the electric field effects known to influence enzyme catalysis,^71^ suggesting that dispersion polarization may play a previously underappreciated role in modulating functions and binding of proteins. The mutual coupling between electrostatics and dispersion, especially in charged environments involving Asp, Glu, Lys, Arg, or solvated ions, is further amplified by electric fields, which can enhance dispersion forces by up to 35%.^78^ As such, understanding this interplay is essential for realistic modeling of biomolecular systems.

From a methodological standpoint, the MBD@FCO model provides physically grounded, nonempirical dispersion energies along with physically consistent corrections to the electron density. This makes it a compelling approach for extending density-functional approximations toward more accurate, correlation-aware treatments of noncovalent interactions. However, coupling such models with existing DFAs remains nontrivial. A persistent challenge is the double-counting of correlation at intermediate ranges when dispersion corrections are added to semilocal functionals. Current damping approaches often rely on empirical parameters, obscuring physical interpretation.^54–56^ Promising solutions include the use of “dispersionless” DFAs,^53,79,80^ or density-dependent range separation schemes based on fitting to high-accuracy reference densities, e.g., from CCSD. An appropriate integration of a “SAPT-consistent” practical method like MBD@FCO with DFAs would enable fully self-consistent calculations, seamlessly accounting for dispersion effects both in energies and densities.

Another open question is how to define rigorous reference densities for dispersion-induced deformations. While the CCSD–HF density difference serves as a reliable benchmark in dispersion-dominated systems at long-range, it is generally contaminated by nondispersion effects, especially in polar or hydrogen-bonded systems. A more rigorous alternative is offered by SAPT, which, in principle, can isolate the density contribution due to dispersion across all distances. Recent work by Tyrcha et al.^81^ shows how to extract induction-induced density shifts from SAPT, providing a promising route for formal decomposition of density distortions. Extending this to dispersion remains an open and promising direction for future research.

Despite these challenges, our work opens new avenues for practical application. MBD@FCO densities can be used to estimate ESP landscape changes due to dispersion interactions. In addition, combining them with nonself-consistent promolecular densities—widely used in biomolecular NCI analysis—might improve interpretability of weak interactions in large systems, such as proteins. This approach requires only atomic coordinates and atomic volume ratios (e.g., from Hirshfeld partitioning^51,82^ or charge population analysis^83^) as input, and can be implemented via postprocessing of standard quantum chemistry outputs using open-source tools such as libMBD.^60^

In summary, this work provides a practical and physically consistent link between dispersion energy calculations and electron density analysis. By demonstrating that vdW-induced density polarization is not only real but might be functionally relevant in large, complex systems, we offer a new perspective on the role of long-range electron correlation in molecular modeling. Our results pave the way for improved chemical interpretation, enhanced visualization tools, and future developments of next-generation “dispersion-consistent” density functionals. Our findings motivate further research into the subtle and cooperative interplay between various noncovalent interactions in increasingly large systems.

## Methods

To compute the vdW polarization density, MBD model as implemented in the libMBD library^60^ was employed. The MBD method maps atoms onto a model Hamiltonian of charged quantum harmonic (Drude) oscillators (QDO) centered at **R**_*A*_, with frequencies ω_*A*_, charges *q*_*A*_ and masses *m*_*A*_. The MBD Hamiltonian for a system of *N* coupled oscillators reads

$${Ĥ}_{\mathrm{MBD}}=\underset{A}{\sum }\left[-\frac{1}{2}\nabla_{\xi_{A}}^{2}+\frac{1}{2}\omega_{A}^{2}\xi_{A}^{2}\right]+\frac{1}{2}\underset{A\neq B}{\sum }\omega_{A}\omega_{B}\sqrt{\alpha_{0,A}\alpha_{0,B}}\xi_{A}T_{\mathrm{AB}}\xi_{B}$$ 6

where $\xi_{A}=\sqrt{m_{A}}\left(r_{A}-R_{A}\right)$ are mass-weighted displacements of the oscillating charges, and **T**_*AB*_ is the dipole interaction tensor. The static polarizabilities α_0,*A*_ and dispersion coefficients *C*_6,*A*_ of atoms are connected to the oscillator parameters via simple relations

7 $$C_{6,A}=\frac{3}{4}\hbar \omega_{A}\alpha_{0,A}^{2},,\alpha_{0,A}=\frac{q_{A}^{2}}{m_{A}\omega_{A}^{2}}$$

Since the MBD Hamiltonian represents a quadratic form, it can be exactly diagonalized, leading to the emergence of 3*N* collective oscillation modes with the frequencies ω̃_*k*_. The MBD energy is then expressed as the change in the zero-point energy of fluctuations due to the dipole interaction

8 $$E_{\mathrm{MBD}}=\sum_{k=1}^{3N}\frac{{\mathrm{\omega ̃}}_{k}}{2}-\sum_{A=1}^{N}\frac{3\omega_{A}}{2}$$

Dipole coupling tensor **T**_*AB*_ defines the interactions between fluctuating dipoles of oscillators and determines the behavior of the MBD model with changing distance. In this work, **T**_*AB*_ was derived from the screened Coulomb interaction of two oscillator (Gaussian) charge densities with widths σ_*A*_^2^ at distance **R**

9 $$\begin{array}{l} T_{\mathrm{AB}}^{\mathrm{ij}}\left(R,\sigma \right)=\frac{\partial^{2}}{\partial R_{i}\partial R_{j}}\frac{\mathrm{erf}\left(\zeta \right)}{R}=\left(\mathrm{erf}\left(\zeta \right)-\Theta \left(\zeta \right)\right)T_{\mathrm{bare}}^{\mathrm{ij}}\left(R\right)+2\zeta^{2}\Theta \left(\zeta \right)\frac{R_{i}R_{j}}{R^{5}}\\ \Theta \left(\zeta \right)=\frac{2\zeta }{\sqrt{\pi }}e^{-\zeta^{2}},\;\zeta =\frac{R}{\sqrt{\sigma_{A}^{2}+\sigma_{B}^{2}}}\left(i,j=x,y,z\right) \end{array}$$

Here, **T**_bare_^*ij*^(**R**) = (−3*R*_*i*_ *R*_*j*_ + δ_*ij*_ *R* ^2^)/*R* ^5^ is the “bare” dipole tensor. The oscillator width is derived from the zero-distance limit of the classical dipole–dipole interaction^34,84^

10 $$\sigma_{A}={\left(\frac{1}{3}\sqrt{\frac{2}{\pi }}\alpha_{0,A}\right)}^{1/3}$$

QDOs were parametrized using the recently proposed vdW-OQDO scheme.^41,50^ In this scheme, the three parameters {ω, *q*, *m*} are determined solely from {α_0_, *C*_6_} by the solution of the system of three equations, two of which are displayed above in eq 7, while the third one stems from the quantum-mechanical connection between the vdW radius and static polarizability.^85,86^ Please see Section S2.3 of the Supporting Information for more details.

All DFT calculations were performed using the Perdew–Burke–Ernzerhof (PBE)^61^ functional as implemented in all-electron FHI-aims code,^87^ with “tight” basis sets and integration grids. The Kohn–Sham self-consistent cycle was converged to 10^–6^ eV in energy and 10^–5^ electrons in density. The input α_0_ and *C*_6_ for the MBD Hamiltonian were obtained from the rescaling of the reference free-atom values with the ratio of Hirshfeld volumes of a free-atom and atom-in-molecule^51^ computed at the PBE level

11 $$\alpha_{0,A}=\alpha_{0,A}^{\mathrm{free}}\left(\frac{V_{A}^{\mathrm{AIM}}}{V_{A}^{\mathrm{free}}}\right),\;C_{6,A}=C_{6,A}^{\mathrm{free}}{\left(\frac{V_{A}^{\mathrm{AIM}}}{V_{A}^{\mathrm{free}}}\right)}^{2}$$

CCSD and HF calculations of electronic density were carried out in the PySCF^88^ code employing augmented correlation-consistent Dunning basis sets^89^ with counterpoise correction. The frozen-core approximation was adopted in CCSD calculations. Additional CCSD and CCSDT calculations were performed using MRCC,^90^ and Q-Chem^91^ was used as a third benchmark for CCSD densities, including orbital relaxation effects. More technical details on CCSD and HF calculations are presented in Section S4 of the Supporting Information.

## Supplementary Material



## Notes

Derivation of MBD density polarization and electrostatic potential; additional results for small molecular dimers, PAHs, and biologically relevant examples; extended coupled-cluster and SAPT reference benchmarks; computational details of CCSD–HF and NCI calculations (PDF)
