# Correction to “Noncovalent Interactions in Density Functional Theory: All the Charge Density We Do Not See”

**DOI:** 10.1021/jacs.6c05929

**Published:** 2026-04-03

**Authors:** Almaz Khabibrakhmanov, Matteo Gori, Carolin Müller, Alexandre Tkatchenko

The authors wish to add the following Data Availability statement to the published article.

## Data Availability

The authors make available the complete data set underlying this work at Zenodo: 10.5281/zenodo.18865966. The repository contains volumetric electron density difference data, electrostatic potentials, numerical data sets, and example scripts used to generate the results presented in the paper.

