# Supplementary material for: Noncovalent Interactions in Density Functional Theory: All the Charge Density We Do Not See
Source: J Am Chem Soc. 2025 Oct 23;147(44):40763–75. doi: 10.1021/jacs.5c13706 (PMC12593390; doi:10.1021/jacs.5c13706)
Supplement: Supplementary file 1 [file ja5c13706_si_001.pdf]

Supporting Information: Noncovalent Interactions in Density Functional Theory: All the Charge Density We Do Not See

Almaz Khabibrakhmanov, Matteo Gori, Carolin Müller, and Alexandre Tkatchenko*

Department of Physics and Materials Science, University of Luxembourg, L-1511 Luxembourg City, Luxembourg

E-mail: alexandre.tkatchenko@uni.lu

This PDF file includes:

Section S1. Supporting Findings

Section S2. MBD Density and Electrostatic Potential

Section S3. CCSD–HF as a Reliable Reference Method

Section S4. CCSD–HF Calculations: Computational Details

Section S5. NCI Analysis: Computational Details

Figures S1 to S15

Tables S1 to S4

S1 Supporting Findings

S1.1 MBD@FCO Energies for the S66 Dataset

Figure S1a shows the dispersion energies for the S66 dataset[1] as computed by MBD@FCO, MBD@rsSCS, and XDM. Similar to the S12L results, DFA-fitted methods MBD@rsSCS and XDM massively underestimate the dispersion energy by about 60-70 %. In contrast, MBD@FCO predictions are much closer to the SAPT-DFT reference,[2] although there is a systematic overbinding by roughly 16 %.

Interestingly, smaller S66 dimer seem to be more challenging than the larger S12L complexes for predicting dispersion energies – all three methods perform consistently worse for S66 (cf. Figure 1 in the main text). Nevertheless, the total DFA+vdW interaction energies are in a perfect agreement with the CCSD(T)/CBS reference (Figure S1b). This is another manifestation that at equilibrium distances, empirically damped dispersion energies

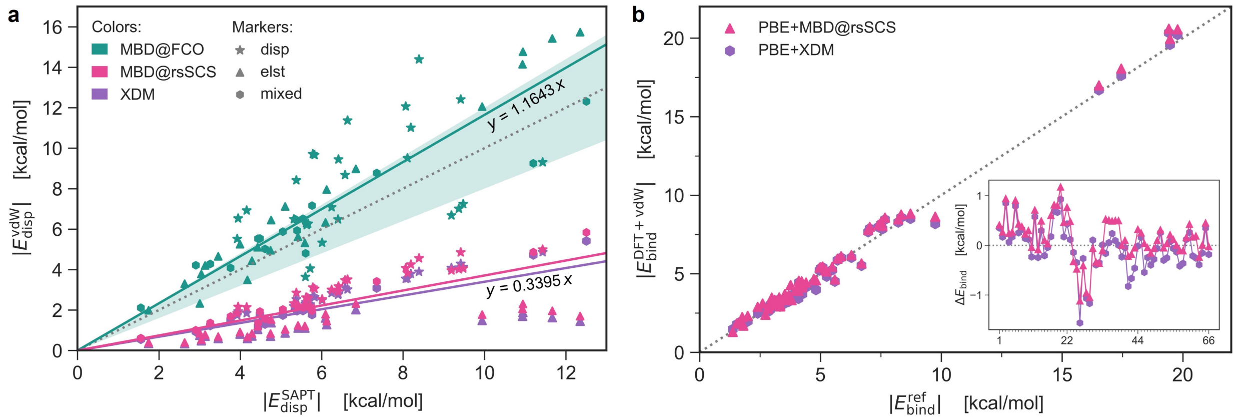

Figure S1. MBD@FCO dispersion energies for the S66 dataset. (**a**) Parity plot of the dispersion energy magnitudes $E_{\mathrm{disp}}^{(2)}$ as computed by SAPT-DFT[2] versus E_{vdW} from the MBD@FCO, MBD@rsSCS and XDM methods. The solid lines display linear fits of data points, and the gray dotted line marks the perfect correlation, with the shaded area highlighting ±20 % interval around. Different markers denote the S66 dimers dominated by dispersion (stars), electrostatics (triangles), or mixed interactions (hexagons), while the colors refer to the vdW methods (see the legend). The linear regression equation for MBD@rsSCS (pink line) is $y = 0.3705x$ (not shown for clarity). (**b**) Parity plot of the total DFA+vdW interaction energy magnitude from PBE+MBD@rsSCS and PBE+XDM methods versus the reference CCSD(T)/CBS energies.[1] The gray dotted line corresponds to the perfect correlation, while the insets show the error $\Delta E = E_{\mathrm{DFA+vdW}} - E_{\mathrm{ref}}$ resolved per system.

have little physical sense, rather serving as a term compensating an error of the underlying DFA.[3–5]

S1.2 CCSD–HF results for small molecular dimers

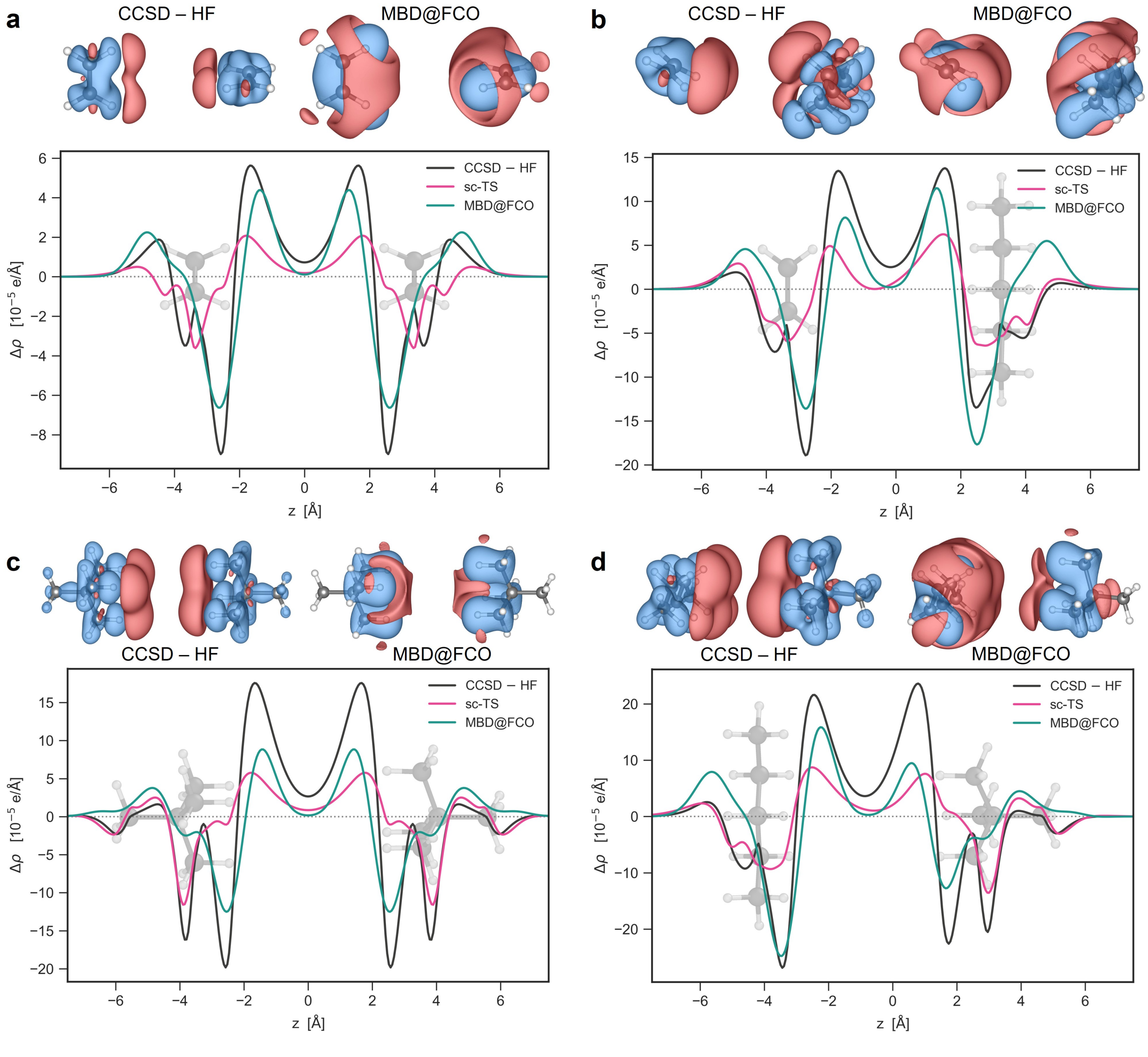

Figure S2. Benchmark against coupled-cluster calculations. Same as Figure 2 in the main manuscript, but for dimers of (**a**) ethene, (**b**) ethene-pentane, (**c**) neopentane, and (**d**) pentane-neopentane. The isovalues of (**a, d**) 4×10^{-6} and (**b, c**) $5 \times 10^{-6}\, e/\text{Å}^3$ were used for visualization.

The six dispersion-dominated dimers were considered, including methane (CH_4), pentane (C_5H_{12}), ethene (C_2H_4), neopentane (C_5H_{12}), ethene-neopentane and neopentane-pentane.

The results for the latter four are displayed in Figure S2 and support our conclusions and analysis reported at the examples of methane and pentane dimer in the main text.

In Figure S3a, we present the vdW-displaced charges computed for the six dimers using CCSD–HF, sc-TS and MBD@FCO. Note the remarkable agreement between MBD@FCO and the reference CCSD–HF. Figure S3b shows the linear correlation between the MBD@FCO displaced charge and the MBD@FCO interaction energy for the S12L and L7+ datasets. This further supports using $Q_{\rm vdW}$ as a measure of dispersion interactions. Interestingly, three out of four outliers in Figure S3b are electrostatically-dominated complexes 6a, 6b and 7a from the S12L dataset.

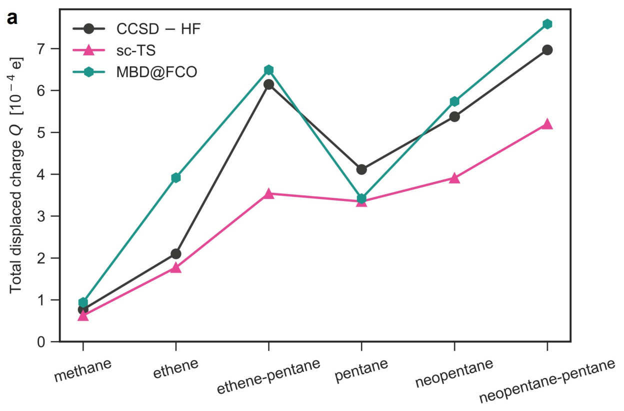

Figure S3. vdW-displaced charge in various systems. (**a**) The results for the six small dimers as computed by the CCSD–HF, sc-TS and MBD@FCO methods. The dimers are ordered by the number of electrons. (**b**) The linear correlation between $Q_{\rm MBD}$ and the MBD@FCO interaction energy $E^{\rm int}_{\rm MBD}$ for the S12L and L7+ datasets. The outliers are displayed, with the ratio of dispersion and electrostatics from SAPT-DFT[6] indicated when available.

S1.3 Results with the Hartree-Fock and DFA Densities

In this section, we present the results for charge displacements in selected systems as calculated by the HF method and several DFAs. Figure S4a,b shows the ratio of vdW-displaced charge $Q_{\rm vdW}$ to its PBE and HF counterparts, $Q_{\rm PBE}$ and $Q_{\rm HF}$, for the six selected systems. Overall, $Q_{\rm HF}$ is about 10 % larger than $Q_{\rm PBE}$, which modifies the ratio accordingly. Nevertheless, this does not change the conclusions made in the main text based on the $Q_{\rm vdW}/Q_{\rm PBE}$

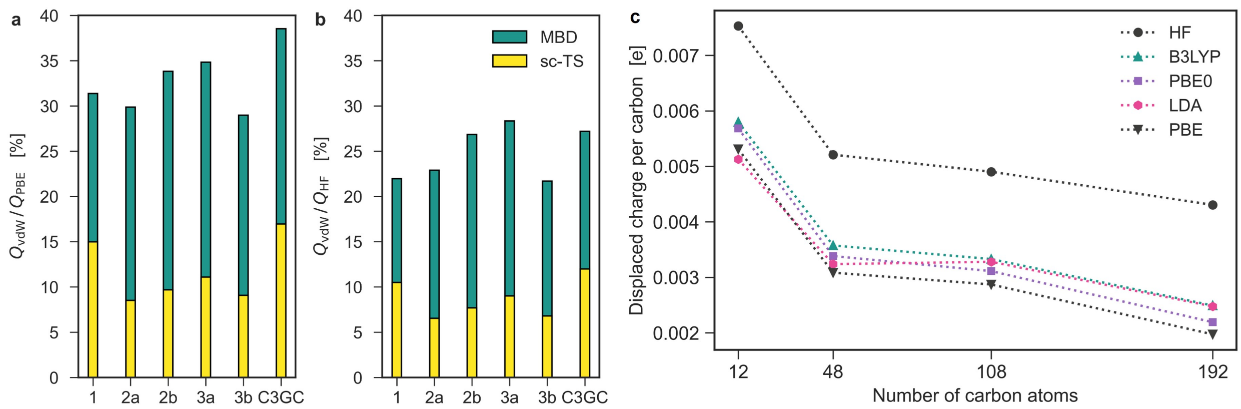

Figure S4. Charge displacement predictions with the HF method and DFAs. (**a,b**) The ratio of vdW-displaced charge Q_{vdW} as computed by sc-TS and MBD methods to (**a**) PBE-displaced charge Q_{PBE} and (**b**) HF-displaced charge Q_{HF} for the six selected systems. (**c**) The system size dependence of total displaced charge in the C1–C4 sequence of PAHs as computed by various mean-field methods.

ratios.

For C1–C4 sequence of polyaromatic hydrocarbons (PAH), the additional calculations of charge displacement were performed with LDA, PBE0 and B3LYP functionals as well as with the Hartree-Fock (HF) method. The results obtained (Figure S4c) witness that both (semi-)local and hybrid DFAs exhibit saturation of displaced charge moving from C1 to C4, and this is not specific to the PBE functional. Thus, all mean-field methods agree in predicting the dimisnishing density distortion due to induction in growing π-conjugated systems.

To show that the effect of vdW-induced polarization on the NCI analysis are also not specific to the choice of DFA, we performed test calculations using the HF densities. The HF and HF+MBD results displayed in Figure S15 manifest essentially the same differences as in the case of PBE and PBE+MBD (Figure 7 of the main text), supporting our point.

S1.4 Additional details on PAH calculations

To explore how vdW density polarization scales with system size, we considered a sequence of polyaromatic hydrocarbon (PAH) dimers from the L7+ dataset: benzene ($C_{12}H_{24}$,

C1C1PD), coronene ($C_{48}H_{24}$, C2C2PD), circumcoronene ($C_{108}H_{36}$, C3C3PD), and circum-circumcoronene ($C_{192}H_{48}$, C4C4PD). In addition, we included intermediate-size dimers of naphthalene ($C_{20}H_{16}$), ovalene ($C_{64}H_{28}$) and hexabenzocoronene ($C_{84}H_{36}$). The graphite-like geometry of naphthalene dimer was taken from Ref. 7, while the geometries of ovalene and hexabenzocoronene were optimized using PBE+MBD@rsSCS following the same protocol as for C3C3PD and C4C4PD.

S1.5 Interacting protein and lipid fragments

In addition to the systems studied in the main text, we considered two example structures representative of interactions between protein and lipid fragments.

The first system is a dimer of two folded polyalanyne (Ala15) chains. The folded chain geometry was obtained from the MD simulations with the SO3LR machine-learning force field.[8] The chain was copied and shifted by 10 Å to form a dimer, which was subsequently optimized in FHI-AIMS using PBE+MBD@rsSCS/'tight' until the 0.01 eV/Å force convergence was reached.

The second example is a diacylglycerol dimer, serving as a simplified model of a lipid. The monomer was obtained from a lipid structure sampled by SO3LR by removing the lipid head and attaching hydrogens instead. It was then copied to make a dimer and preoptimized by SO3LR, which was followed by the PBE+MBD@rsSCS/'tight' geometry optimization with the 0.01 eV/Å force convergence threshold.

For these two systems, we computed the MBD@FCO density polarization and evaluated the MBD- and PBE-displaced charges. The atomic structures, MBD@FCO density isosurfaces and displaced charge values are shown in Figure S5. In the Ala15 dimer, we obtained the ratio $Q_{\mathrm{vdW}}/Q_{\mathrm{PBE}} = 20\,\%$, which demonstrates that vdW density polarization can be significant also in protein fragment interactions. Moreover, we consider this as a lower bound and expect a stronger effect in more realistic proteins containing various amino acid residues, including more polarizable aromatic ones.

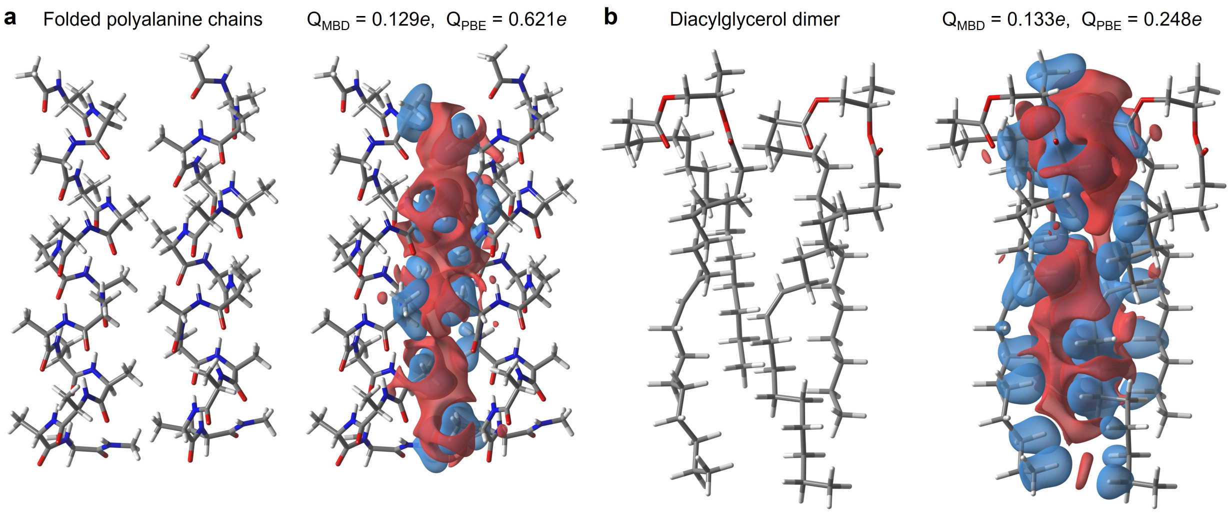

Figure S5. vdW density polarization in prototype protein and lipid systems. (**a**) Atomic structure (left) and 3×10^{-5} a.u. isosurface of MBD@FCO density shift (right) for the dimer of two folded Ala15 chains. The MBD@FCO and PBE displaced charges are also displayed. (**b**) The same as (**a**) but for the diacylglycerol dimer.

In the diacylglycerol case, the ratio $Q_{\text{vdW}}/Q_{\text{PBE}}$ is about 50 % due to long alkane chains as a part of the structure. This allows us to inspect the effect of symmetry on the $Q_{\text{vdW}}/Q_{\text{PBE}}$ ratio in alkane chains, by comparing to a highly symmetric example of octadecane dimer (CBH) from the main text. While we note that $Q_{\text{vdW}}/Q_{\text{PBE}}$ ratio in diacylglycerol is lower, the vdW density polarization is still comparable to the PBE induction effects.

S2 MBD Density and Electrostatic Potential

S2.1 MBD Density Polarization

In this section, we derive the explicit analytical expression for MBD density polarization from Eq. (1) of the main text. The ground state wave function for a system of N non-interacting quantum Drude oscillators is a Gaussian state of the form

$$\Psi_0(\{\mathbf{r}_A\}) = \langle \{\mathbf{r}_A\} | \Psi_0 \rangle = \prod_{A=1}^{N} \left(\frac{m_A \omega_A}{\hbar} \right)^{3/4} \exp\left[-\frac{m_A \omega_A}{2\hbar} (\mathbf{r}_A - \mathbf{R}_A)^2 \right] . \tag{S1}$$

The ground state of the MBD Hamiltonian is fully described by the $3N \times 3N$ symmetric positive-definite correlation matrix $\mathbf{C}$ between QDOs dipole fluctuations, which is defined as:

$$\mathbf{C} = \hbar^{-1}\, \mathbf{M}\mathbf{O}^T \widetilde{\mathbf{\Omega}} \mathbf{O}\mathbf{M}\,, \tag{S2}$$

where $\mathbf{M} = \mathrm{diag}\{\sqrt{m_1}, \sqrt{m_1}, \sqrt{m_1}, ..., \sqrt{m_N}, \sqrt{m_N}, \sqrt{m_N}\}$ is the matrix of the square root of QDO masses, $\mathbf{O}$ is the $3N \times 3N$ orthogonal matrix diagonalizing the quadratic MBD potential, i.e.

$$\widetilde{\mathbf{\Omega}}^2 = \mathrm{diag}\{\tilde{\omega}_1^2, ..., \tilde{\omega}_{3N}^2\} = \mathbf{O}(\mathbf{\Omega}^2 + \mathbf{V}_{\mathrm{dip}})\mathbf{O}^T\,, \tag{S3}$$

with $[\mathbf{V}_{\mathrm{dip}}]_{AB} = \omega_A \omega_B \sqrt{\alpha_{0,A}\alpha_{0,B}}\; \mathbf{T}_{AB}$ being the 3×3 dimensional block of dipole interaction matrix. Correlation matrix can also be viewed as composed of 3×3 blocks $\mathbf{C}_{AB}$, and with this notation the MBD ground-state wavefunction $\Psi(\{\mathbf{r}_A\}) = \langle\{\mathbf{r}_A\}|\Psi\rangle$ can be written as

$$\Psi(\{\mathbf{r}_A\}) = \left[\left(\prod_{A=1}^{N} m_A^3\right)\left(\prod_{\alpha=1}^{3N} \frac{\tilde{\omega}_\alpha}{\hbar}\right)\right]^{1/4} \exp\left[-\frac{1}{2}\sum_{A,B=1}^{N} (\mathbf{r}_A - \mathbf{R}_A)^T \mathbf{C}_{AB} (\mathbf{r}_B - \mathbf{R}_B)\right]. \tag{S4}$$

Eq. (1) of the main text for the vdW-induced density polarization reads

$$\rho_{\mathrm{pol}}(\mathbf{r}) = \sum_{A=1}^{N} q_A \left[\int\limits_{\mathbb{R}^{3N}} \left(|\Psi(\{\mathbf{r}_A\})|^2 - |\Psi_0(\{\mathbf{r}_A\})|^2\right) \delta(\mathbf{r} - \mathbf{r}_A)\ \mathrm{d}^3\mathbf{r}_1 \ldots \mathrm{d}^3\mathbf{r}_N\right]. \tag{S5}$$

Substituting Eqs. (S1) and (S4) and performing the integration as in Refs. 9–11, one can obtain:

$$\rho_{\mathrm{pol}}(\mathbf{r}) = \sum_{A=1}^{N} \frac{q_A}{\pi^{3/2}} \left(\frac{\exp\left[-(\mathbf{r} - \mathbf{R}_A)^T \mathbf{K}_{AA} (\mathbf{r} - \mathbf{R}_A)\right]}{\Lambda_A^3} - \frac{\exp\left[-\lambda_A^{-2}(\mathbf{r} - \mathbf{R}_A)^2\right]}{\lambda_A^3}\right), \tag{S6}$$

where $\lambda_A = \sqrt{\hbar/m_A\omega_A}$ and $\Lambda_A^3 = \sqrt{\det \mathbf{C}^{/A}/\det \mathbf{C}}$ are constant prefactors, and $\mathbf{K}_{AA}$ is a 3×3 matrix obtained from the correlation matrix as

$$\mathbf{K}_{AA} = \mathbf{C}_{AA} - \mathbf{C}_A^T\,[\mathbf{C}^{/A}]^{-1}\,\mathbf{C}_A\,. \tag{S7}$$

Here, $\mathbf{C}_{AA}$ is the A-th diagonal block of $\mathbf{C}$. The $(3N-3)\times(3N-3)$ square matrix $\mathbf{C}^{/A}$ is obtained from $\mathbf{C}$ by removing from it a tri-row vector $\mathbf{C}_A^T$ sized $3\times(3N-3)$ and a tri-column vector $\mathbf{C}_A$ sized $(3N-3)\times 3$, which correspond to matrix indices $\{A, A+1, A+2\}$. Note that $\mathbf{C}_A^T$ and $\mathbf{C}_A$ do not include the diagonal elements of $\mathbf{C}$, which enter the $\mathbf{C}_{AA}$ matrix. Finally, $[\mathbf{C}^{/A}]^{-1}$ denotes the inverse of $\mathbf{C}^{/A}$ matrix. Figure S6 schematically explains the introduced notation.

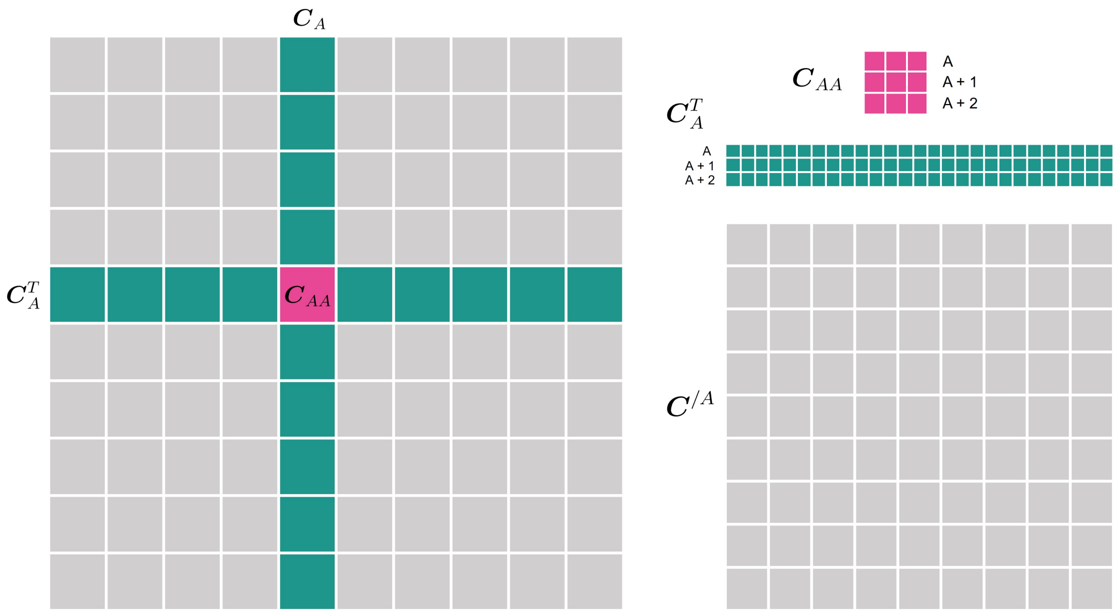

Figure S6. Schematic illustration of the notation for MBD correlation matrix.

S2.2 Connection to electron density

The derived polarization density can be used to refine the underlying KS electron density $\rho(\mathbf{r})$. We introduce the total wave function and the total density operator for the KS+MBD

system as

$$\Phi = \Psi_{\mathrm{KS}}\big(\{\mathbf{r}_i\}\big)\cdot\Psi_{\mathrm{MBD}}\big(\{\mathbf{r}_A\}\big) , \quad \hat{\rho}_{\mathrm{tot}} = \hat{\rho}_{\mathrm{elec}} + \hat{\rho}_{\mathrm{MBD}} = \sum_{i=1}^{N_{\mathrm{elec}}} e\delta(\mathbf{r}-\hat{\mathbf{r}}_i) + \sum_{A=1}^{N} q_A\delta(\mathbf{r}-\hat{\mathbf{r}}_A) , \quad \text{(S8)}$$

where $\Psi_{\mathrm{KS}}\big(\{\mathbf{r}_i\}\big)$ is the Slater determinant of KS orbitals, depending on coordinates of *real* electrons, and $\Psi_{\mathrm{MBD}}\big(\{\mathbf{r}_A\}\big)$ is the MBD wave function, depending on coordinates of *drudons* – quasiparticles, mimicking collective correlated response of valence electrons in an atom. It is important to emphasize that the MBD Hamiltonian does not directly depend on real electronic coordinates; in fact, the dipole-coupled oscillators represent an auxiliary model quantum system assisting in capturing the intricate electron correlations in a coarse-grained way. Moreover, for a given system there are N_{elec} electrons and N (equal to the number of atoms) drudons, with all electrons carrying elementary charge e and each oscillator having, in general, a different charge q_A (see Figure S7). Hence, $\hat{H}_{\mathrm{MBD}}$ and $\hat{H}_{\mathrm{KS}}$ can be viewed as existing in distinct Hilbert spaces, and the total wave function can be represented as a product state.

The total charge density of the KS+MBD system is straightforward to evaluate as

$$\rho_{\mathrm{tot}}(\mathbf{r}) = \langle\Phi|\hat{\rho}_{\mathrm{tot}}|\Phi\rangle = \langle\Psi_{\mathrm{KS}}|\hat{\rho}_{\mathrm{elec}}|\Psi_{\mathrm{KS}}\rangle + \langle\Psi_{\mathrm{MBD}}|\hat{\rho}_{\mathrm{MBD}}|\Psi_{\mathrm{MBD}}\rangle = \rho_{\mathrm{KS}}(\mathbf{r}) + \rho_{\mathrm{MBD}}(\mathbf{r}) . \quad \text{(S9)}$$

Since the MBD Hamiltonian uses a harmonic bond between drudon and its nuclei, it cannot model the full charge density reasonably. Nevertheless, as we show in this work, the oscillators can model *changes* in density due to the vdW dispersion. Therefore, to compute electrostatic potentials and the NCI isosurfaces we use $\rho_{\mathrm{tot}}(\mathbf{r}) = \rho_{\mathrm{PBE}}(\mathbf{r}) + \rho_{\mathrm{pol}}(\mathbf{r})$, with $\rho_{\mathrm{pol}}(\mathbf{r})$ defined in Eq. (S6).

The optimal choice of charges q_A within the MBD model is essential for accurate density predictions. Using the vdW-OQDO parametrization, we assign species-specific charges based on α_0 and C_6 coefficients, in contrast to the earlier MBD approaches using arbitrary $q_A = 1$ a.u. charge values.[12,13] Figure S7 shows the histogram of these charges for the S12L dataset.

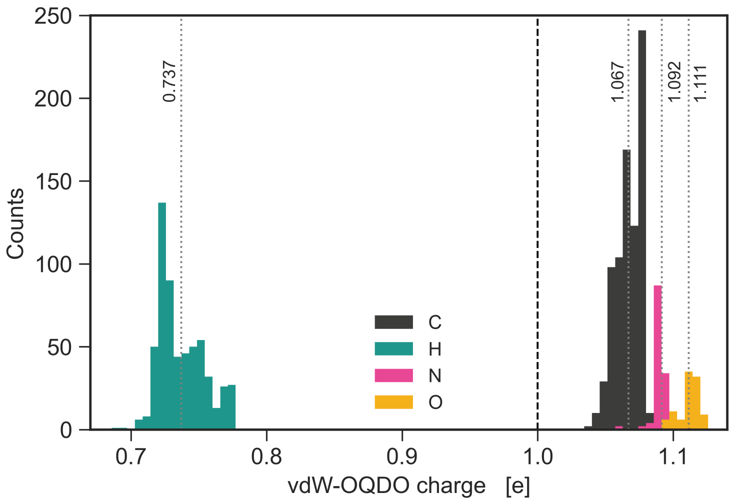

Figure S7. Histogram of vdW-OQDO charge across the S12L dataset. For every species, the dotted gray line denotes the position of the mean value (displayed in the plot). In the conventional parametrization of MBD@rsSCS, all charges are set to 1 a.u., which is highlighted by the gray dashed line.

S2.3 Parametrization of the MBD@FCO Model

The conventional parametrization of oscillators in the MBD model[12,14] reads (formulas in this section use SI units):

$$\omega = \frac{4}{3}\frac{C_6}{\hbar \alpha_0^2 k_e^2} \ , \quad m = \frac{1}{\alpha_0 \omega^2} \ , \quad q = \sqrt{\alpha_0 m \omega^2} \equiv 1 \text{ a.u.} \ , \tag{S10}$$

with $k_e = 1/4\pi\varepsilon_0$. In this work, the recently proposed optimal vdW-OQDO parametrization scheme[15,16] was applied. It can be summarized as follows:

$$\omega = \frac{4}{3}\frac{C_6}{\hbar \alpha_0^2 k_e^2} \ , \quad m = \frac{\hbar x_L}{\omega r_B^2} \ , \quad q = \sqrt{\alpha_0 m \omega^2} \ . \tag{S11}$$

Here, $r_B = 4\pi\varepsilon_0\hbar^2/m_e e^2$ is the Bohr radius, and x_L is the largest among the two roots of the following dimensionless transcendental equation:

$$a e^{bx} = 2x^2 + \frac{x}{b} \ , \quad x = \frac{\mu \omega r_B^2}{\hbar} \ , \tag{S12}$$

with constants a and b defined as

$$a = \frac{9\alpha_{\text{fsc}}^{4/3}}{64}\ ,\quad b = \frac{2(\alpha_0/4\pi\varepsilon_0)^{2/7}}{\alpha_{\text{fsc}}^{8/21} r_B^{6/7}}\ , \tag{S13}$$

and $\alpha_{\text{fsc}} = e^2/4\pi\varepsilon_0\hbar c \approx 1/137.036$ being the fine-structure constant.

S2.4 Electrostatic Potential Calculations

Electrostatic potential $\varphi(\mathbf{r})$ created at point $\mathbf{r}$ by the charge density $\rho(\mathbf{r})$ is given by

$$\varphi(\mathbf{r}) = -\int \frac{\rho(\mathbf{r}')}{|\mathbf{r}' - \mathbf{r}|}\ d^3\mathbf{r}'\ . \tag{S14}$$

The chosen sign convention corresponds to a positive test charge and is consistent with most quantum-chemistry codes, such as Q-Chem. However, a plus sign convention is also possible, as implemented e.g. in FHI-aims, and $\varphi(\mathbf{r})$ then corresponds to the Hartree potential for electrons. So, additional sign correction must be made before cross-checking results from these two codes.

To compute the contribution to ESP from the MBD polarization density, in Eq. (S14) we put $\rho = \rho_{\text{pol}}$ from Eq. (S6). Since $\lambda_A^{-2}(\mathbf{r} - \mathbf{R}_A)^2 = \lambda_A^{-2}(\mathbf{r} - \mathbf{R}_A)^T\mathbf{I}(\mathbf{r} - \mathbf{R}_A)$, with $\mathbf{I}$ being the identity matrix, to get the ESP from ρ_{pol} one needs to calculate generic integrals of the form:

$$J_A(\mathbf{r}, \mathbf{Y}) = \int \frac{\exp\left[-(\mathbf{r}' - \mathbf{R}_A)^T\mathbf{Y}(\mathbf{r}' - \mathbf{R}_A)\right]}{|\mathbf{r}' - \mathbf{r}|}\ d^3\mathbf{r}'\ , \tag{S15}$$

where $\mathbf{Y}$ is a symmetric positive definite matrix. Introducing $\boldsymbol{\xi} = \mathbf{r}' - \mathbf{R}_A$ and $\mathbf{r}_A = \mathbf{r} - \mathbf{R}_A$, we obtain:

$$J_A = \int \frac{e^{-\boldsymbol{\xi}^T\mathbf{Y}\boldsymbol{\xi}}}{|\boldsymbol{\xi} - \mathbf{r}_A|} d^3\boldsymbol{\xi} = \frac{2}{\sqrt{\pi}} \int_0^\infty ds \int d^3\boldsymbol{\xi}\ \exp\left[-\boldsymbol{\xi}^T\mathbf{Y}\boldsymbol{\xi}\right] \times \exp\left[-s^2(\boldsymbol{\xi}^2 - 2\mathbf{r}_A^T\boldsymbol{\xi} + \mathbf{r}_A^2)\right]\ , \tag{S16}$$

where we used the integral representation of the Coulomb potential:

$$\frac{1}{|\mathbf{x}|} = \frac{2}{\sqrt{\pi}} \int\limits_0^\infty ds \, \exp(-s^2 \mathbf{x}^2) \, . \tag{S17}$$

By doing a coordinate shift $\boldsymbol{\eta} = \boldsymbol{\xi} - s^2 \left[\mathbf{Y}'(s)\right]^{-1} \mathbf{r}_A$,[1] where $\mathbf{Y}'(s) = \mathbf{Y} + s^2\mathbf{I}$, and completing the square in the exponent, we arrive at

$$J_A = \frac{2}{\sqrt{\pi}} \int\limits_0^\infty ds \int d^3\boldsymbol{\eta} \; \exp\left[-\boldsymbol{\eta}^T \mathbf{Y}'(s) \boldsymbol{\eta}\right] \times \exp\left[s^4 \mathbf{r}_A^T [\mathbf{Y}'(s)]^{-1} \mathbf{r}_A - s^2 \mathbf{r}_A^2\right] \, . \tag{S18}$$

The Gaussian integral over $\boldsymbol{\eta}$ gives $\pi^{3/2} \left[\det \mathbf{Y}'(s)\right]^{-1/2}$, leading to

$$J_A(\mathbf{r}, \mathbf{Y}) = 2\pi \int\limits_0^\infty ds \; \left[\det \mathbf{Y}'(s)\right]^{-1/2} \exp\left[\mathbf{r}_A^T \mathbf{Q}(s) \mathbf{r}_A\right] \, , \tag{S19}$$

where we defined the new matrix $\mathbf{Q}(s) = s^4[\mathbf{Y}'(s)]^{-1} - s^2\mathbf{I} = s^4[\mathbf{Y} + s^2\mathbf{I}]^{-1} - s^2\mathbf{I}$. The integrand function has a highly non-linear dependence on s through the determinant of $\mathbf{Y}'(s)$, which prevents the fully analytical evaluation. However, the integrand function behaves smoothly and quickly decays, which enables efficient evaluation of the integral using a numerical quadrature. The standard `quad_vec` function from the `SciPy` library was used for computing quadratures. Testing this function against manually implemented Gauss-Legendre quadrature with 15 points revealed agreement up to 6 digits.

Thus, the final expression for the MBD ESP reads:

$$\varphi_{\mathrm{MBD}}(\mathbf{r}) = -\int \frac{\rho_{\mathrm{pol}}(\mathbf{r}')}{|\mathbf{r}' - \mathbf{r}|} \, d^3\mathbf{r}' = \sum_{A=1}^{N} \frac{q_A}{\pi^{3/2}} \left[\frac{J_A(\mathbf{r}, \mathbf{K}_{AA})}{\Lambda_A^3} - \frac{J_A(\mathbf{r}, \mathbf{I})}{\lambda_A^3}\right] \, . \tag{S20}$$

To plot ESP maps, we evaluated Eq. (S20) on a cube grid and further used ChimeraX[17] software to produce images. To focus on the long-range ESP, the $2R_{\mathrm{vdW}}$ surfaces were

[1] This can always be done, since matrix $\mathbf{Y}'(s)$ is symmetric positive definite and hence invertible.

modeled as solvent-accessible surfaces with a probe radius of 1.4 Å (the default value) and twice increased vdW radii of all atoms. For the buckyball catcher, the ESP at the PBE level was computed using Q-Chem with aug-cc-pVDZ basis sets. For the protein ESP, we used FHI-aims with 'tight' basis sets for efficiency reasons.

S2.5 Validation of the MBD@FCO electrostatic potential

To illustrate robustness of the MBD@FCO method for predicting the long-range ESP shifts due to dispersion, we benchmark it against the CCSD–HF/aug-cc-pVDZ ESP difference at the example of a parallel-displaced benzene dimer (Figure S8). The ESP maps the $2R_{\mathrm{vdW}}$ surface obtained using CCSD–HF and MBD@FCO are in excellent agreement. This is further

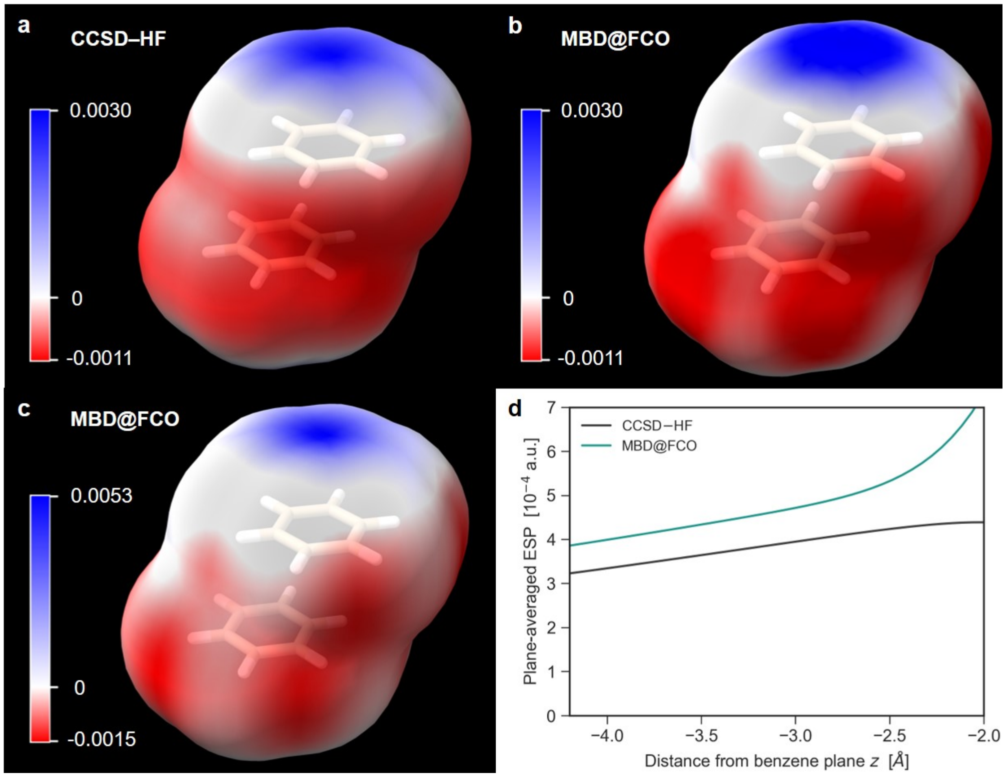

Figure S8. Electrostatic potential of a benzene dimer. (**a, b**) ESP maps (in a.u.) on the $2R_{\mathrm{vdW}}$ surface of the dimer as computed by the CCSD–HF/aug-cc-pVDZ and MBD@FCO. The colorbar scaled to the CCSD–HF ESP range was used to create both images. (**c**) The ESP map (in a.u.) from MBD@FCO with the appropriately normalized (per maximum and minimum values) colorbar scale. (**d**) The tails of the plane-averaged ESP plotted along z-axis of the dimer for the two methods.

supported by the plane-averaged ESP plot, with the long-range decay of MBD@FCO ESP tails closely matching the CCSD–HF reference (Figure S8d). We remark that at distances shorter than 3 Å, MBD@FCO might overestimate ESP, as its soft harmonic potential is parametrized to match the long-range atomic response properties. Nevertheless, this benchmark validates MBD@FCO as a reliable method to predict the long-range ESP in aromatic systems, as done in Figures 5 and 6 of the main text.

S3 CCSD–HF as a Reliable Reference Method

S3.1 Effect of coupled-cluster triples on density

As a reference for vdW density polarization $\Delta\rho$, we use the double difference between coupled-cluster singles and doubles (CCSD) and Hartree-Fock (HF) levels of theory,

$$\Delta\rho_{\mathrm{CCSD-HF}}(\mathbf{r}) = \left[\rho^{\mathrm{D}}_{\mathrm{CCSD}}(\mathbf{r}) - \rho^{\mathrm{M1}}_{\mathrm{CCSD}}(\mathbf{r}) - \rho^{\mathrm{M2}}_{\mathrm{CCSD}}(\mathbf{r})\right] - \left[\rho^{\mathrm{D}}_{\mathrm{HF}}(\mathbf{r}) - \rho^{\mathrm{M1}}_{\mathrm{HF}}(\mathbf{r}) - \rho^{\mathrm{M2}}_{\mathrm{HF}}(\mathbf{r})\right] \ , \tag{S21}$$

which captures polarization of density due to the (long-range) electronic correlation effects at *ab initio* level. Although CCSD is not chemically accurate for energies (often worse than MP2), this method is widely applied as a reference for electron density in closed-shell systems.[18–20] Why CCSD densities are "more accurate" than CCSD energies can be understood e.g. from the so-called Wigner's $2n+1$ rule in many-body perturbation theory (MBPT).[21] This rule states that the $2n$-th and/or $(2n+1)$-th order perturbation contributions to a non-degenerate energy can be obtained from a knowledge of the wavefunction through n-th order.[21] In other words, having e.g. MP2 wave function is sufficient to obtain MP4-level energies. Similar considerations are valid in coupled cluster theory,[21,22] and hence the CCSD wave function (and the density) can be considered as effectively having access to beyond-CCSD energies.

Nevertheless, to examine the effect of triples, essential to obtain chemical accuracy for

correlation energies, we performed calculations of the coupled-cluster density including full triples (CCSDT)[2] for methane and neon dimers. The CCSDT densities were shown to be almost identical to the composite CCSDTQ densities for atoms, ions, and small molecules,[23] and therefore CCSDT can be considered as a highly accurate reference method for densities.

The obtained CCSDT–HF density differences for methane and neon dimers show only minor quantitative changes compared to the CCSD–HF counterparts (Figure S9). For example, by evaluating displaced charges (as defined in the main manuscript) we found that in methane dimer, $Q_{\mathrm{CCSD-HF}}$ is only 12 % smaller than $Q_{\mathrm{CCSDT-HF}}$. This validates using CCSD as the reference method for systems larger than methane or neon when CCSDT becomes prohibitively expensive.

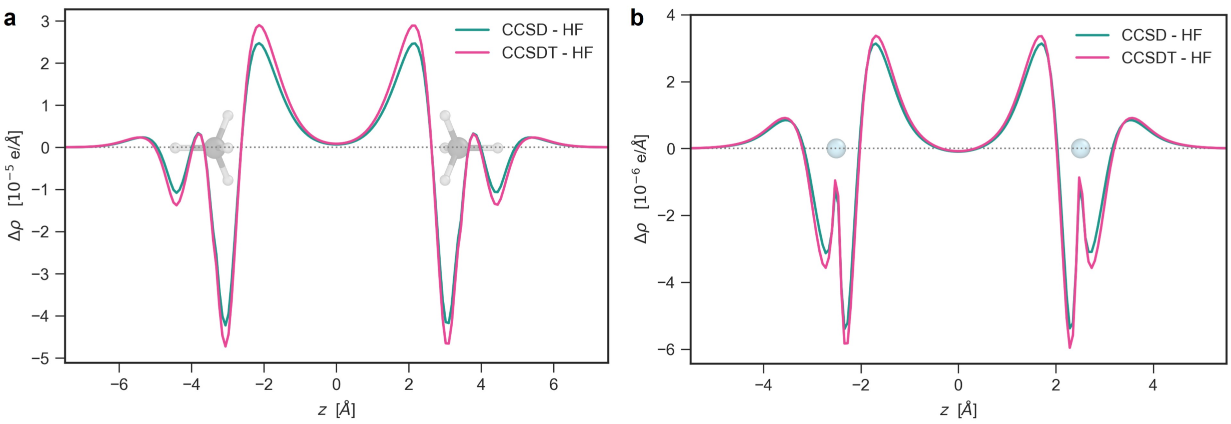

Figure S9. Effect of including full coupled-cluster triples on density. Benchmark of CCSD–HF versus CCSDT–HF for **(a)** methane and **(b)** neon dimers. Calculations were performed using aug-cc-pVDZ basis set in the MRCC code.[24]

S3.2 Effect of the mean-field reference method

The choice of the mean-field reference also influences the resulting density polarization. From the quantum chemistry point of view, the HF method by definition provides a rigorous uncorrelated reference for coupled cluster methods. However, we should note that the CCSD–HF difference is generally not equivalent to dispersion-induced polarization and contains

[2]We computed CCSDT densities, since CCSD(T) gradients are not widely implemented in quantum chemistry programs.

also other effects, arising e.g. due to the differences between CCSD and HF description of induction interaction. We illustrate this in Figure S10 and Table S1 at the contrasting examples of methane and benzene dimers. We consider HF, PBE, and PBE0 as the mean-field references, and compute the respective differences with CCSD densities.

In methane dimer, the long-range interaction is strongly dominated by vdW dispersion, hence the CCSD–HF difference is almost exclusively due to dispersion-induced polarization. This is also supported by the analysis of quadrupole moments (Table S1), which shows that there are no significant differences between HF, PBE0, and CCSD for the change in quadrupole moment due to intermonomer interactions. PBE and PBE0 functionals spill excessive charge into intermolecular space compared to CCSD, which leads to negative $\Delta\rho$ (Figure S10a) and is represents a manifestation of delocalization error. Therefore, the HF method seems to be the most reasonable reference method for the methane dimer.

This is in sharp contrast with parallel-displaced benzene dimer (at 6 Å distance), where CCSD–HF, CCSD–PBE, and CCSD–PBE0 produce qualitatively different density deformation profiles (Figure S10b). Compared to CCSD, PBE functional again spills too much

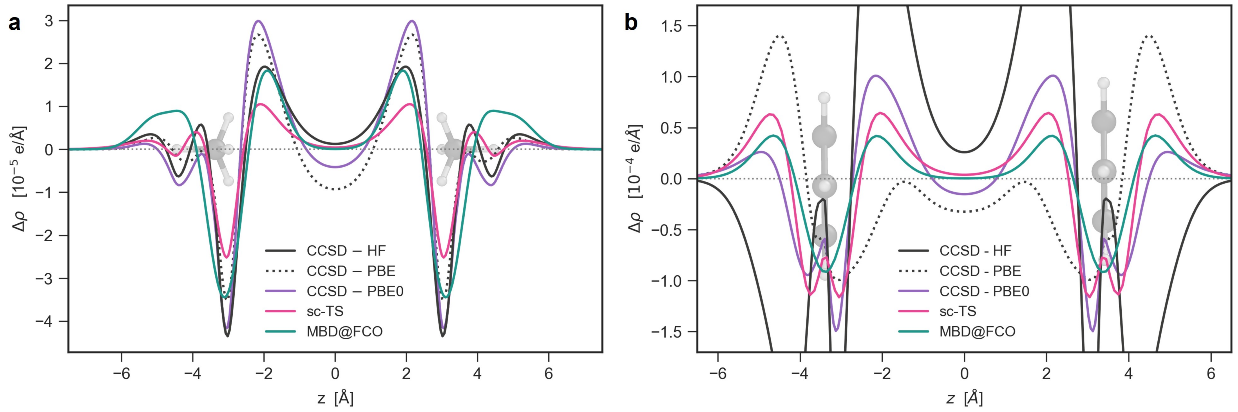

Figure S10. Effect of the mean-field reference method on density polarization in **(a)** methane and **(b)** benzene dimers. The plots show plane-averaged density polarization $\Delta\rho(z)$ as computed using CCSD, HF, PBE and PBE0 methods in PySCF with **(a)** aug-cc-pVQZ and **(b)** aug-cc-pVDZ basis sets, as well as $\Delta\rho(z)$ calculated with the sc-TS and MBD@FCO methods (see the text for further explanations). The monomer geometries are shown along the distance axis to scale. In the case of benzene **(b)**, the extrema values for CCSD–HF are 3.17×10^{-4} and -2.95×10^{-4} e/Å (not shown in the plot for clarity).

Table S1. Quadrupole moments of methane and benzene dimers as computed in Q-CHEM with aug-cc-pVQZ and aug-cc-pVDZ basis sets, respectively. The HF, PBE0, and CCSD values (in Debye · Å) of Q_{zz} component of quadrupole moment tensor (in Cartesian representation) are reported for the dimers and their monomers, together with the differences (in bold).

methane	HF	PBE0	CCSD
$Q_{\mathrm{zz}}^{\mathrm{mono}}$	−8.4125	−8.3976	−8.3905
$Q_{\mathrm{zz}}^{\mathrm{dimer}}$	−16.8453	−16.8175	−16.8003
$Q_{\mathrm{zz}}^{\mathrm{dimer}} - 2Q_{\mathrm{zz}}^{\mathrm{mono}}$	**−0.0203**	**−0.0223**	**−0.0193**

benzene	HF	PBE0	CCSD
$Q_{\mathrm{zz}}^{\mathrm{mono}}$	−41.2886	−39.9845	−40.7897
$Q_{\mathrm{zz}}^{\mathrm{dimer}}$	−83.4515	−80.7073	−82.2708
$Q_{\mathrm{zz}}^{\mathrm{dimer}} - 2Q_{\mathrm{zz}}^{\mathrm{mono}}$	**−0.8743**	**−0.7383**	**−0.6914**

charge into intermolecular space (negative $\Delta\rho$), which is transferred from the outer sides of the monomers (positive peaks in $\Delta\rho$). The HF density is, in contrast, too localized on monomers, which leads to overly large magnitudes of $\Delta\rho$ outside monomers. Among the considered mean-field methods, hybrid PBE0 functional seems to deliver the most balanced description of benzene dimer's density for our purposes.

These findings can be rationalized by the analysis of quadrupole moments (Table S1), which shows a good agreement between PBE0 and CCSD for $Q_{\mathrm{zz}}^{\mathrm{dimer}} - 2Q_{\mathrm{zz}}^{\mathrm{mono}}$ (7 % error), while the HF predictions are substantially worse (26 % error). This suggests that in the case of benzene dimer, CCSD–HF density difference is contaminated with the effects beyond vdW dispersion interactions, while the CCSD–PBE0 deformation density seems to be much closer to the dispersion-induced density deformation, which is highlighted by a good agreement with the sc-TS and MBD@FCO methods (Figure S10b).

We note that these findings do not contradict using CCSD–HF as the reference for ESP in section S2.5, because ESP is calculated using the total density of the dimer, without subtracting monomers. Hence, CCSD–HF difference is an adequate measure for the contribution of electronic correlations to the ESP in that case.

To sum it up, filtering out the density deformation due to the vdW dispersion interactions using *ab initio* methods is a challenging task. In principle, an alternative approach would be considering the dispersion-induced wave function correction from SAPT. However,

at the time of writing, such implementations are not yet available. Therefore, for small dispersion-dominated dimers, which we use to benchmark the MBD@FCO method, we opt for the CCSD–HF method as the reference for density deformations. In the next section, we corroborate this by energy-based arguments.

S3.3 Benchmarking CCSD–HF energies against SAPT

It is well known that for getting chemical accuracy, perturbative triples are essential to include in coupled-cluster calculations, in particular for dispersion interactions. Here, we tested how well CCSD captures the dispersion energy in systems where we benchmark the densities. We consider a set of eight dispersion-dominated dimers (see the full list in Table S2) from the S22×5[25] and S66×8 datatsets.[1] To ensure that interaction energies are not contaminated with non-dispersion contributions, we used the geometries at twice larger separations than in equilibrium (available at `begdb.org`).

First, we benchmarked CCSD counterpoise-corrected interaction energies against the reference CCSD(T)/CBS values from Ref. 1. The energies from the two methods correlate very well but CCSD misses about 14 % of the interaction energy in average (Figure S11a). We note that the mean-field methods – PBE and HF – fail badly in predicting interaction energies in these correlation-bound systems, with PBE being slightly better.

Next, we benchmark the energy differences between the coupled cluster and mean-field methods against the accurate SAPT2(+3) total dispersion energies from Li *et al.*[26] (including exchange-dispersion and corrections due to intramonomer correlations). CCSD(T)–HF difference shows almost ideal agreement with SAPT, with the average error under 3 %.[3] CCSD–HF misses about 16 % of the dispersion energy, consistent with the findings in Figure S11a. This also correlates well with the 12 % less displaced charge predicted by CCSD–HF relative

[3]We note that CCSD(T)–HF differences were computed using the reference CCSD(T)/CBS energies from Ref. 1 and the HF energies computed by ourselves in PySCF with a given basis set (see Table S2). The same basis sets were used to get the CCSD energies. Thus, there might be a small error due to a finite basis set size; however, we estimate it to be very small for energy differences, and hence it should not affect our conclusons.

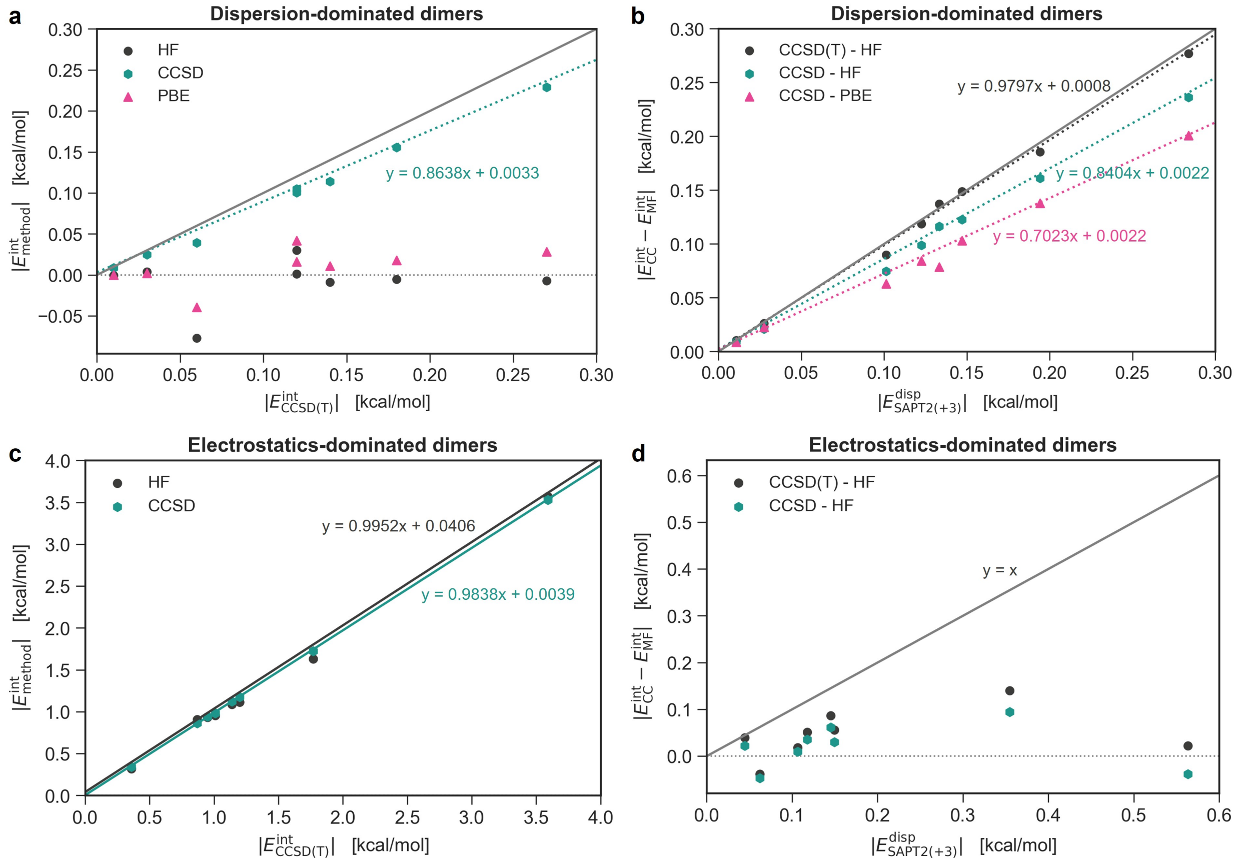

Figure S11. Benchmark of CC–HF energies versus SAPT2(+3) dispersion energies. (**a**) Parity plot of HF, CCSD and PBE interaction energies against the reference CCSD(T)/CBS values[1] for 8 dispersion-dominated dimers. (**b**) Parity plot of coupled cluster minus mean-field interaction energies versus the SAPT2(+3) dispersion energies[26] for 8 dispersion-dominated dimers. (**c**) The same as (**a**) but for 8 electrostatics-dominated dimers. (**d**) The same as (**b**) but for 8 electrostatics-dominated dimers. Gray solid lines denote perfect correlation, while colored lines show linear regression fits to the data.

to CCSDT–HF (see Section S3.1). CCSD–PBE underbinds by 30 % and shows slightly larger scatter of values around the mean line. This is explained by the fact that PBE captures a small part of dispersion energy, while HF is purely dispersion-free. Hence, the difference CCSD–HF is closer to the true dispersion energy than CCSD–PBE and correspondingly, the CCSD–HF density difference should be used as a reference for dispersion-induced polarization in these systems.

Unfortunately, for non-dispersion-bound systems, the CCSD–HF difference cannot be treated as such a reference. We illustrate this by considering a set of eight electrostatics-

Table S2. **The molecular dimers used in coupled cluster versus SAPT benchmark.**

Dispersion-dominated			Electrostatics-dominated		
**Dimers**	**Dataset**	**Basis**	**Dimers**	**Dataset**	**Basis**
methane	S22×5	aQZ	water (#1)	S66×8	aDZ
ethene	S22×5	aTZ	water-MeOH (#2)	S66×8	aTZ
benzene-methane	S22×5	aDZ	water-MeNH2 (#3)	S66×8	aTZ
benzene (#24)	S66×8	aDZ	MeOH (#5)	S66×8	aDZ
ethene-pentane (#44)	S66×8	aTZ	MeNH2-water (#12)	S66×8	aTZ
pentane (#34)	S66×8	aDZ	peptide (#15)	S66×8	aDZ
neopentane-pentane (#35)	S66×8	aDZ	AcOH (#20)	S66×8	aTZ
neopentane (#36)	S66×8	aDZ	ammonia	S22×5	aTZ

dominated dimers (see the full list in Table S2). For consistency, we used the geometries at twice larger separations than in equilibrium, just like above. But in contrast to the previous case, even the HF method performs well in predicting their interaction energies, being very close to CCSD and CCSD(T), with a small systematic error of 0.04 kcal/mol. Comparison against SAPT dispersion energies reveals that the CCSD–HF differences cannot be associated with dispersion, as well as CCSD(T)–HF. Thus, currently there is no theoretically justified reference available for dispersion-induced densities in such systems. Potentially, this could be resolved by SAPT-based approaches similar to the recent work by Tyrcha *et al.*[27]

S4 CCSD–HF Calculations: Computational Details

S4.1 Basis set convergence

CCSD and HF calculations of electron density were carried out in PySCF (v.2.6.2)[28] employing augmented correlation-consistent Dunning basis sets[29] with counterpoise correction and frozen-core approximation adopted for CCSD. For the methane dimer, we performed a cross-validation of the PySCF results with Q-Chem (v.6.1)[30] and MRCC,[24] and the perfect agreement was obtained between the two codes (Figure S12a,b), ensuring the reproducibility of our results. In addition, we examined basis set convergence of the CCSD−HF density differences by performing calculations with aug-cc-pVXZ basis sets, where X = D, T, Q for

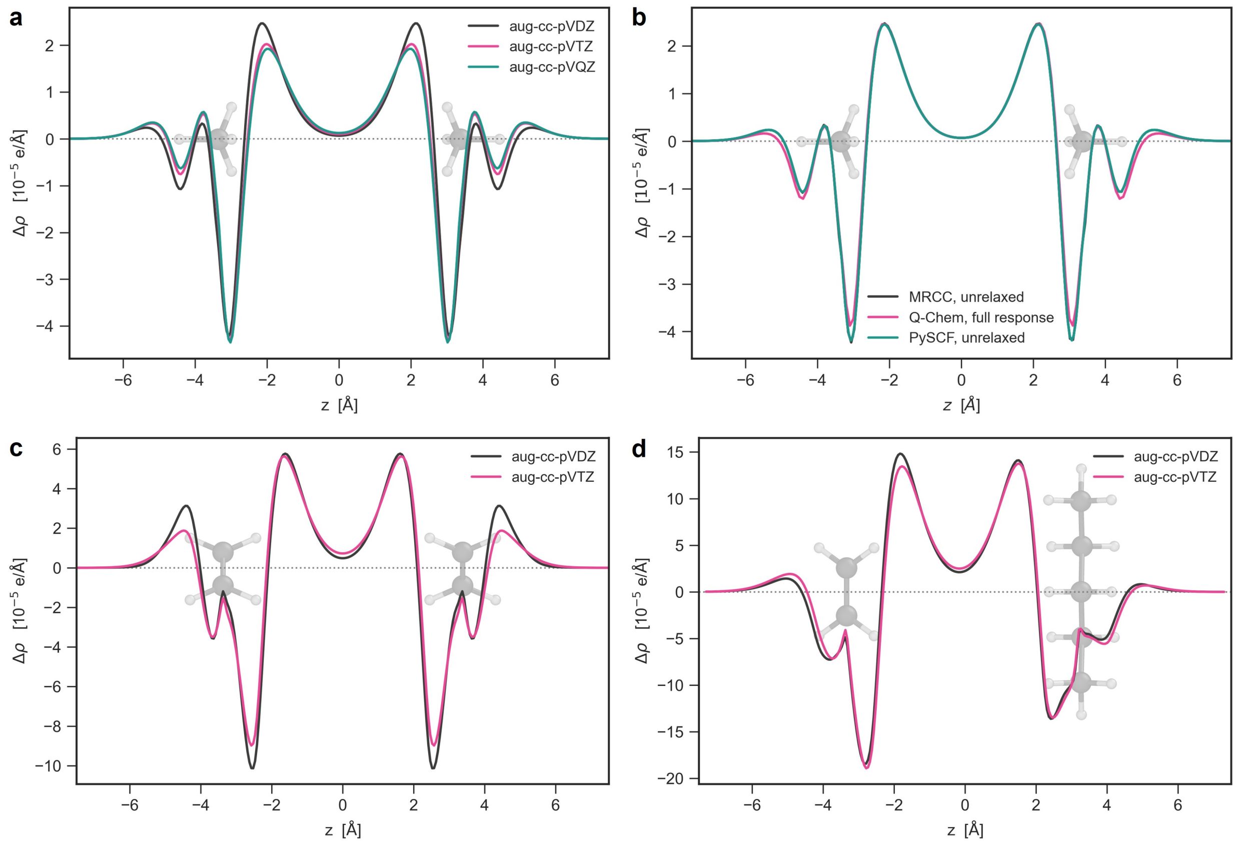

Figure S12. Convergence tests for $\Delta\rho_{\mathrm{CCSD-HF}}$ calculations. **(a)** Basis set convergence for methane dimer. **(b)** Effect of orbital relaxation in CCSD density calculations and benchmark of PySCF, Q-Chem and MRCC codes. **(c)** Basis set convergence for ethene dimer. **(d)** Basis set convergence for ethene-pentane dimer.

methane dimer and X = D, T for ethene and ethene-pentane dimers (Figure S12). The observed variations are rather small in all three cases. In methane, TZ basis gives practically the same result as QZ, while DZ only slightly overpolarizes the dimer. For ethene and ethene-pentane dimer, DZ basis also shows the close agreement with TZ, which was the largest affordable therein. Overall, our results prove that aug-cc-pVDZ basis set already gives sufficiently converged results, which justifies its use for pentane and neopentane dimers. Table S3 summarizes the information about the systems and basis sets used for production calculations.

For benzene dimer, CCSD/aug-cc-pVDZ calculations were performed in PySCF. For naphthalene and coronene dimers, the CCSD/jul-cc-pVDZ calculations of density were per-

formed by Prof. Péter R. Nagy using the pre-release version of MRCC optimized for computing CCSD gradients. The calculation for coronene dimer took 46 hours on 2 nodes with 112 cores per node (i.e., 10k core hours). The system contains 108 correlated orbitals, and the memory consumption reached 222 GB/MPI task (there were 4 MPI tasks in total). All these make it the largest CCSD gradient computation ever done, at least to our best knowledge.

Table S3. The basis sets used in CCSD and HF production calculations.

Dimer	N_{elec}	Basis set	N_{basis}
methane	20	aug-cc-pVQZ	528
ethene	32	aug-cc-pVTZ	368
ethene-pentane	58	aug-cc-pVTZ	690
pentane-pentane	84	aug-cc-pVDZ	446
pentane-neopentane	84	aug-cc-pVDZ	446
neopentane-neopentane	84	aug-cc-pVDZ	446
benzene	84	aug-cc-pVDZ	384
naphthalene	136	jul-cc-pVDZ	540
coronene	312	jul-cc-pVDZ	1224

S4.2 Sensitivity to density precision

The calculations of plane-averaged $\Delta\rho_{\text{CCSD}-\text{HF}}(z)$ have to be done using electron densities represented on a real-space grid in the `.cube` format; therefore, the sufficient numerical precision of data in these files has to be provided. The default `.cube` printing precision in PySCF is 5 digits after the comma, which was observed to lead to a numerical noise when computing $\Delta\rho_{\text{CCSD}-\text{HF}}(z)$ (Figure S13). It was found that this noise increases with increasing basis set cardinality and system size. We revealed that outputting the density to the `.cube` file with 9 digits precision eliminates the noise, and the source code of PySCF (`cubegen.py` file) was modified accordingly. This setting was used for all PySCF calculations. For comparison, the default `.cube` printing precision is 12 digits in Q-Chem and 8 digits in FHI-aims. The cuboid grid spacing was set to 0.1 Bohr to ensure the sufficient sampling of the density.

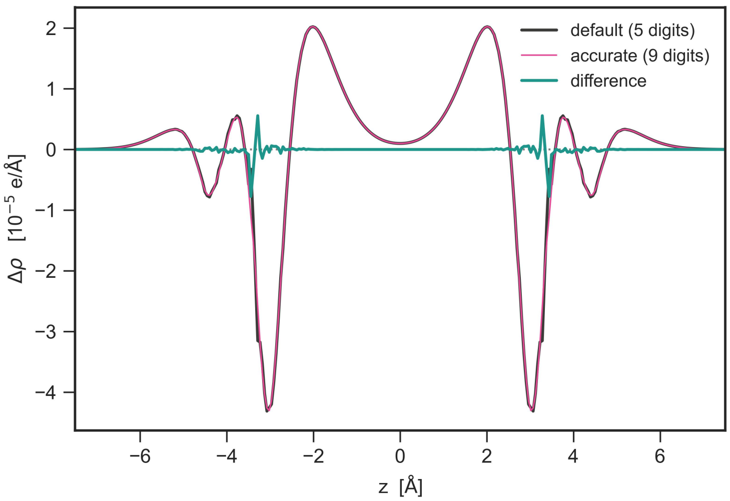

Figure S13. Effect of the numerical precision on plane-averaged density difference. $\Delta\rho_{\mathrm{CCSD-HF}}(z)$ for methane dimer (aug-cc-pVTZ basis) with different `.cube` file precision.

S5 NCI Analysis: Computational Details

The density-based code NCImilano[31] was used NCI analysis of PBE and PBE+MBD densities. The code was modified by its author Dr. Gabriele Saleh upon our request to make it compatible with the FHI-aims `.cube` formatting. NCImilano uses the 4th-order finite-difference formulas to compute derivatives of electron density[31] and also evaluates volume and charge of NCI isosurfaces within the fixed range of $s_{\mathrm{iso}} = 0.1 - 1.0$.

The densities computed using the PBE functional in FHI-aims were output in the `.cube` 3D grid format. Analytical MBD density polarization from Eq. (S6) was evaluated on the same 3D grid using the self-written Python script relying on the libMBD[32] library. The total PBE+MBD densities were obtained as a sum of PBE and MBD densities on the grid. The grids had a step of $h = 0.1$ Å and covered the whole system plus 7 Bohrs of empty space as measured from the outermost atoms of the system.

While often rather fine grids are employed for NCI analysis (0.05–0.1 Bohr and less),[31,33] for large systems this explodes `.cube` file size, making the following analysis time- and resource-consuming. The chosen $h = 0.1$ Å spacing was found to be a fair compromise delivering sufficiently accurate representation of density and its derivatives while maintaining

Table S4. Insensitivity of NCI volume ratios to grid spacing. Ratio $\gamma = V_{\text{NCI}}^{\text{PBE+MBD}}/V_{\text{NCI}}^{\text{PBE}}$ for selected systems computed using grids with 0.1 and 0.05 Å step.

Systems	$h = 0.1$ Å	$h = 0.05$ Å
benzene	1.950	2.026
7b ADOH-CB7	2.669	2.663
C3GC	2.180	2.210

manageable file size. This was ensured by test calculations employing twice finer grids with $h = 0.05$ Å, in which no significant differences both in NCI isosurface plots and NCI volume ratios were observed compared to 0.1 Å step size, as summarized in Table S4.

To focus on non-covalent interactions, charge density cutoffs exclude covalent bonds and regions near nuclei, typically using $\rho_c = 0.05$ a.u.[33,34] NCI isosurfaces are defined over an s_{iso} range, usually $0.2 - 0.7$, with the specific value chosen to match NCI signatures from the 2D plots (see Figure S14a). An example NCI isosurface for the 7b host-guest complex, using $s_{\text{iso}} = 0.5$, is displayed in the inset, demonstrating the correspondence between the $s(\rho)$ diagram and the 3D NCI isosurface.

To evaluate the ratio $\gamma = V_{\text{NCI}}^{\text{PBE+MBD}}/V_{\text{NCI}}^{\text{PBE}}$, we used $s_{\text{iso}} = 0.5$ for all systems and found minimal variation in γ within the typical NCI range of $s_{\text{iso}} = 0.4 - 0.6$, leaving overall trends unchanged (see Figure S14b). The results for the S12L and L7+ datasets show substantial increases in NCI volumes across all systems. The largest enhancement is found in alkane chains (CBH, $\gamma = 3.23$), consistent with vdW-driven interactions, while the smallest change occurs in the benzene dimer ($\gamma = 1.95$). The γ ratio shows a weak dependence on system size, increasing only slightly from 1.95 to 2.14 in the C1−C4 sequence of PAHs. On the other hand, γ is sensitive to interaction type, with non-vdW systems like water and formic acid dimers (hydrogen bonds), or bicyclo[2.2.2]octene (steric repulsion) from Ref. 33 exhibiting γ values close to 1. This highlights the MBD@FCO ability to distinguish vdW-driven ($\gamma = 2 - 3$) from non-vdW ($\gamma \sim 1$) interactions.

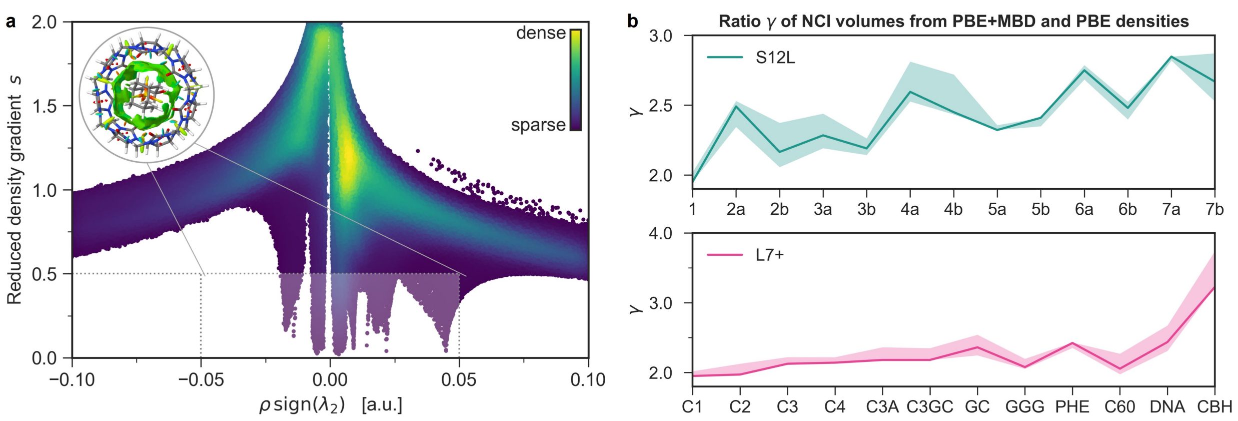

Figure S14. NCI analysis explained. (**a**) Two-dimensional NCI plot computed from PBE+MBD density at the example of the 7b host-guest complex. Color coding indicates the statistical distribution of the data points on the graph: the brighter the color, the more data points are clustered there. The shaded region shows the subset of data points used to generate the NCI isosurface $s_{\text{iso}} = 0.5$ (the inset). (**b**) The ratio γ of NCI isosurface volumes computed from PBE+MBD and PBE densities for S12L (top) and L7+ (bottom) molecular sets. The lines display values computed at $s_{\text{iso}} = 0.5$, and shaded regions indicate the spread of γ when $s_{\text{iso}} = 0.4 - 0.6$.

Finally, our tests using HF density instead of PBE density show that the trends remain the same upon including the MBD@FCO correction (Figure S15), and hence our conclusions are not sensitive to this choice of the mean-field reference method.

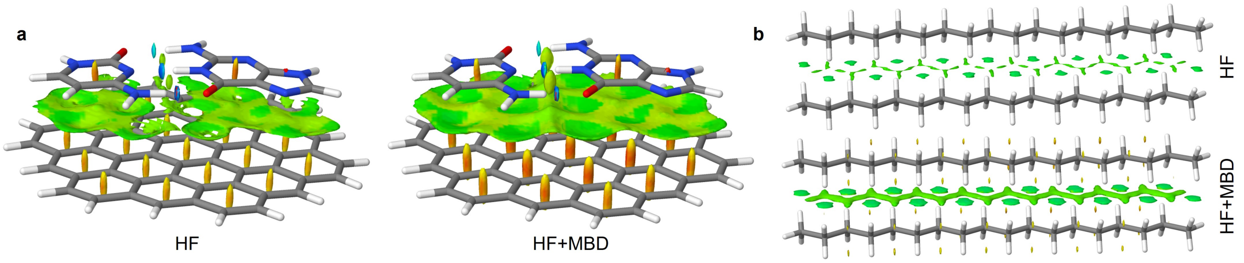

Figure S15. NCI analysis of the HF density with and without vdW contribution. Same as Figure 7 but calculated using HF and HF+MBD@FCO charge densities for (**a**) C3GC (L7+): $s = 0.5$, $[-0.05, 0.04]$; (**b**) CBH (L7+): $s = 0.65$, $[-0.02, 0.02]$.

References

(1) Řezáč, J.; Riley, K. E.; Hobza, P. S66: A well-balanced database of benchmark interaction energies relevant to biomolecular structures. *J. Chem. Theory Comput.* **2011**, *7*, 2427–2438.

(2) Heßelmann, A. DFT-SAPT intermolecular interaction energies employing exact-exchange Kohn–Sham response methods. *J. Chem. Theory Comput.* **2018**, *14*, 1943–1959.

(3) Shahbaz, M.; Szalewicz, K. Do semilocal density-functional approximations recover dispersion energies at small intermonomer separations? *Phys. Rev. Lett.* **2018**, *121*, 113402.

(4) Shahbaz, M.; Szalewicz, K. Evaluation of methods for obtaining dispersion energies used in density functional calculations of intermolecular interactions. *Theor. Chem. Acc.* **2019**, *138*, 25.

(5) Gray, M.; Herbert, J. M. *Annu. Rep. Comput. Chem.*; Elsevier, 2024; Vol. 20; pp 1–61.

(6) Heßelmann, A.; Korona, T. Intermolecular symmetry-adapted perturbation theory study of large organic complexes. *J. Chem. Phys.* **2014**, *141*, 094107.

(7) Janowski, T.; Pulay, P. A Benchmark Comparison of σ/σ and π/π Dispersion: the Dimers of Naphthalene and Decalin, and Coronene and Perhydrocoronene. *J. Am. Chem. Soc.* **2012**, *134*, 17520–17525.

(8) Kabylda, A.; Frank, J. T.; Dou, S. S.; Khabibrakhmanov, A.; Sandonas, L. M.; Unke, O. T.; Chmiela, S.; Müller, K.-R.; Tkatchenko, A. Molecular simulations with a pretrained neural network and universal pairwise force fields. *ChemRxiv: 10.26434/chemrxiv-2024-bdfr0-v3* **2025**,

(9) Hermann, J. Towards Unified Density-Functional Model of van der Waals Interactions. Ph.D. thesis, Humboldt-Universität zu Berlin, 2018.

(10) Stöhr, M. van der Waals Dispersion Interactions in Biomolecular Systems: Quantum-Mechanical Insights and Methodological Advances. Ph.D. thesis, University of Luxembourg, 2020.

(11) Khabibrakhmanov, A. Bridging Quantum Drude Oscillators and Electronic-Structure Theory for van der Waals Dispersion Interactions. Ph.D. thesis, University of Luxembourg, 2025.

(12) Hermann, J.; Alfe, D.; Tkatchenko, A. Nanoscale π–π stacked molecules are bound by collective charge fluctuations. *Nat. Commun.* **2017**, *8*, 14052.

(13) Stöhr, M.; Sadhukhan, M.; Al-Hamdani, Y. S.; Hermann, J.; Tkatchenko, A. Coulomb interactions between dipolar quantum fluctuations in van der Waals bound molecules and materials. *Nat. Commun.* **2021**, *12*, 137.

(14) Tkatchenko, A.; DiStasio Jr, R. A.; Car, R.; Scheffler, M. Accurate and efficient method for many-body van der Waals interactions. *Phys. Rev. Lett.* **2012**, *108*, 236402.

(15) Góger, S.; Khabibrakhmanov, A.; Vaccarelli, O.; Fedorov, D. V.; Tkatchenko, A. Optimized Quantum Drude Oscillators for Atomic and Molecular Response Properties. *J. Phys. Chem. Lett.* **2023**, *14*, 6217–6223.

(16) Khabibrakhmanov, A.; Fedorov, D. V.; Tkatchenko, A. Universal Pairwise Interatomic van der Waals Potentials Based on Quantum Drude Oscillators. *J. Chem. Theory Comput.* **2023**, *19*, 7895–7907.

(17) Pettersen, E. F.; Goddard, T. D.; Huang, C. C.; Meng, E. C.; Couch, G. S.; Croll, T. I.; Morris, J. H.; Ferrin, T. E. UCSF ChimeraX: Structure visualization for researchers, educators, and developers. *Protein Sci.* **2021**, *30*, 70–82.

(18) Ferri, N.; DiStasio Jr, R. A.; Ambrosetti, A.; Car, R.; Tkatchenko, A. Electronic properties of molecules and surfaces with a self-consistent interatomic van der Waals density functional. *Phys. Rev. Lett.* **2015**, *114*, 176802.

(19) Medvedev, M. G.; Bushmarinov, I. S.; Sun, J.; Perdew, J. P.; Lyssenko, K. A. Density functional theory is straying from the path toward the exact functional. *Science* **2017**, *355*, 49–52.

(20) Brorsen, K. R.; Yang, Y.; Pak, M. V.; Hammes-Schiffer, S. Is the accuracy of density functional theory for atomization energies and densities in bonding regions correlated? *J. Phys. Chem. Lett.* **2017**, *8*, 2076–2081.

(21) Kvasnička, V.; Laurinc, V.; Biskupič, S. Wigner's (2 n+ 1) rule in MBPT. *Mol. Phys.* **1980**, *39*, 143–161.

(22) Kucharski, S. A.; Bartlett, R. J. *Advances in Quantum Chemistry*; Elsevier, 1986; Vol. 18; pp 281–344.

(23) Mezei, P. D.; Csonka, G. I.; Kállay, M. Electron density errors and density-driven exchange-correlation energy errors in approximate density functional calculations. *J. Chem. Theory Comput.* **2017**, *13*, 4753–4764.

(24) Kállay, M.; Nagy, P. R.; Mester, D.; Rolik, Z.; Samu, G.; Csontos, J.; Csóka, J.; Szabó, P. B.; Gyevi-Nagy, L.; Hégely, B.; others The MRCC program system: Accurate quantum chemistry from water to proteins. *J. Chem. Phys.* **2020**, *152*, 074107.

(25) Gráfová, L.; Pitoňák, M.; Řezáč, J.; Hobza, P. Comparative study of selected wave function and density functional methods for noncovalent interaction energy calculations using the extended S22 data set. *J. Chem. Theory Comput.* **2010**, *6*, 2365–2376.

(26) Li, A.; Muddana, H. S.; Gilson, M. K. Quantum mechanical calculation of noncovalent

interactions: a large-scale evaluation of PMx, DFT, and SAPT approaches. *J. Chem. Theory Comput.* **2014**, *10*, 1563–1575.

(27) Tyrcha, B.; Gupta, T.; Patkowski, K.; Zuchowski, P. S. Analytical derivatives of symmetry-adapted perturbation theory corrections for interaction-induced properties. *J. Chem. Theory Comput.* **2025**, *21*, 4562–4578.

(28) Sun, Q.; Zhang, X.; Banerjee, S.; Bao, P.; Barbry, M.; Blunt, N. S.; Bogdanov, N. A.; Booth, G. H.; Chen, J.; Cui, Z.-H.; others Recent developments in the PySCF program package. *J. Chem. Phys.* **2020**, *153*, 024109.

(29) Kendall, R. A.; Dunning, T. H.; Harrison, R. J. Electron affinities of the first-row atoms revisited. Systematic basis sets and wave functions. *J. Chem. Phys.* **1992**, *96*, 6796–6806.

(30) Epifanovsky, E.; Gilbert, A. T.; Feng, X.; Lee, J.; Mao, Y.; Mardirossian, N.; Pokhilko, P.; White, A. F.; Coons, M. P.; Dempwolff, A. L.; others Software for the frontiers of quantum chemistry: An overview of developments in the Q-Chem 5 package. *J. Chem. Phys.* **2021**, *155*, 084801.

(31) Saleh, G.; Lo Presti, L.; Gatti, C.; Ceresoli, D. NCImilano: an electron-density-based code for the study of noncovalent interactions. *J. Appl. Crystallogr.* **2013**, *46*, 1513–1517.

(32) Hermann, J.; Stöhr, M.; Góger, S.; Chaudhuri, S.; Aradi, B.; Maurer, R. J.; Tkatchenko, A. libMBD: A general-purpose package for scalable quantum many-body dispersion calculations. *J. Chem. Phys.* **2023**, *159*, 174802.

(33) Johnson, E. R.; Keinan, S.; Mori-Sánchez, P.; Contreras-García, J.; Cohen, A. J.; Yang, W. Revealing Noncovalent Interactions. *J. Am. Chem. Soc.* **2010**, *132*, 6498–6506.

(34) Contreras-García, J.; Johnson, E. R.; Keinan, S.; Chaudret, R.; Piquemal, J.-P.; Beratan, D. N.; Yang, W. NCIPLOT: a program for plotting noncovalent interaction regions. *J. Chem. Theory Comput.* **2011**, *7*, 625–632.
